# Natural variations in a pectin acetylesterase gene, *MdPAE10*, contribute to prolonged apple fruit shelf life

**DOI:** 10.1002/tpg2.20084

**Published:** 2021-02-18

**Authors:** Bei Wu, Fei Shen, Chi Jie Chen, Li Liu, Xuan Wang, Wen Yan Zheng, Yang Deng, Ting Wang, Zhen Yu Huang, Chen Xiao, Qian Zhou, Yi Wang, Ting Wu, Xue Feng Xu, Zhen Hai Han, Xin Zhong Zhang

**Affiliations:** ^1^ College of Horticulture China Agricultural University No. 2 Yuanmingyuan West Road, Haidian District Beijing 100193 China

## Abstract

Room‐temperature shelf life is a key factor in fresh market apple (*Malus domestica* Borkh.) quality and commercial value. To investigate the genetic and molecular mechanism underlying apple shelf life, quantitative trait loci (QTL) were identified using bulked segregant analysis via sequencing (BSA‐seq). Ethylene emission, flesh firmness, or crispness of apple fruit from 1,273 F_1_ plants of *M. asiatica* Nakai ‘Zisai Pearl’ × *M. domestica* ‘Golden Delicious’ were phenotyped prior to and during 6 wk of room‐temperature storage. Segregation of ethylene emission and the flesh firmness or crispness traits was detected in the population. Thirteen QTL, including three major ones, were identified on chromosome 03, 08, and 16. A candidate gene encoding pectin acetylesterase, *MdPAE10*, from the QTL Z16.1 negatively affected fruit shelf life. A 379‐bp deletion in the coding sequence of *MdPAE10* disrupted its function. A single nucleotide polymorphism (SNP) in the *MdPAE10* promoter region reduced its transcription activity. These findings provided insight into the genetic control of fruit shelf life and can be potentially used in apple marker‐assisted selection.

## Core Ideas

- Thirteen confident QTL associating apple fruit shelf life were identified.
- A QTL‐derived *MdPAE10* negatively affects fruit shelf life by enhancing pectin solubilization.
- A 379‐bp deletion in the coding sequence of *MdPAE10* disrupts its function.

**Abbreviations**
1‐MCP: 1‐methylcyclopropene
BSA‐seq: bulked segregant analysis via sequencing
cDNA: complementary DNA
CDS: coding sequence
CEP: chelator‐extractable pectin
InDel: insertion–deletion
KASP: Kompetitive allele‐specific PCR
NEP: Na_2_CO_3_–extractable pectin
PCR: polymerase chain reaction
QTL: quantitative trait loci
SNP: single nucleotide polymorphism
VIGS: virus induced gene silencing
WEP: water‐extractable pectin.

## 1 INTRODUCTION

The softening of fleshy fruit is a critical trait that results in reduced shelf life. Cold chain facilities are used worldwide during packing, shipping, storage, and at the retail phase to maintain the quality of fleshy fruit, especially those such as apple (*Malus domestica* Borkh.) that undergo climacteric ripening. Most apple cultivars gradually lose their firmness starting soon after harvest, but some, such as ‘Red Fuji’, retain flesh firmness for at least a month at room temperature after harvest (Gussman et al., 1993). Breeding for cultivars with better fruit shelf life at ambient temperatures has the potential to reduce production costs substantially by avoiding cold chain conditions and prolonging the time of market availability (Wakasa et al., 2006).

The two fruit texture attributes that determine shelf life—flesh firmness and crispness—are closely correlated but clearly distinct. Consumers prefer crisp but less firm apple cultivars such as ‘Honeycrisp’ (McKay et al., 2011). Both flesh firmness and crispness are known to be quantitatively inherited and linked with multiple quantitative trait loci (QTL) (Costa et al., 2005; Matas et al., 2015). For example, QTL associated with apple fruit firmness or crispness have been mapped to chromosome 01, 08, 10, 15, and 16 (Baumgartner et al., 2015; Costa et al., 2010; Costa et al., 2005; Costa et al., 2008; Harada et al., 2000; Zhu & Barritt, 2008). A total of 22 QTL for mechanical and acoustic fruit texture components have been identified in apple, explaining variance from 10 to 49% (Longhi et al., 2012). Using a Bayesian multi‐QTL pedigree‐based approach, a large number of QTL for apple hypanthium firmness after 2 mo of cold storage were also mapped to chromosome 01, 03, 06, 10, 15, and 16, which was consistent with multigenic inheritance of fruit firmness (Bink et al., 2014). Based on six full‐sib pedigreed families and a collection of 233 apple accessions, pedigree‐based analysis and a genome‐wide association study were employed to decipher the genetic control of fruit texture; chromosome 10 was found to be important in controlling the mechanical properties, and chromosome 02 and 14 were more associated with the acoustic response (Di Guardo et al., 2017). Natural mutations in several functional genes have been identified and validated via QTL‐based gene mining. Molecular markers that mapped to the ethylene biosynthesis genes, 1‐aminocyclopropane‐1‐carboxylic acid (ACC) synthase (*MdACS1*) and ACC oxidase (*MdACO1*), were shown to be independently linked to apple fruit shelf life. Apple cultivars with *MdACS1‐2* and *MdACO1‐1* homozygous genotypes showed superior shelf life (Costa et al., 2005).

Similarly, the expression of ethylene biosynthetic proteins was much lower in high‐firmness phenotypes (Marondedze & Thomas, 2012). During fruit ripening, the expression of *MdACS1*, *MdACS3*, and *MdACS5A* increased rapidly in the fruit cortex of the short‐shelf‐life apple *M. domestica* Borkh. cultivar Golden Delicious but not in the long‐shelf‐life cultivar Fuji. *MdACO1* transcript levels increased in the fruit cortex of both cultivars (Li et al., 2010). Increased expression of the ethylene receptor genes, *MdETR1*, *MdETR2*, *MdERS1*, and *MdERS2* has also been observed in apple fruit cortex during ripening (Li et al., 2010). Mutations that affect apple fruit firmness have also been linked to a group of genes encoding cell‐wall modifying proteins. For example, β‐galactosidase, α‐arabinofuranosidase (α‐AFase), and pectin methylesterase (*MdPME*) play roles in modifying cell‐wall structure and fruit firmness. The expression of these proteins is usually higher in the apple genotypes with softer fruit (Gwanpua et al., 2014; Marondedze & Thomas, 2012). Some highly significant fruit texture QTL have been reported to colocalize with genes involved in cell‐wall metabolism, such as the pectinases polygalacturonase 1 (*MdPG1*) and pectate lyase (*MdPel*), as well as the ripening regulators *MdNOR* and *MdRIN* (Longhi et al., 2012). Downregulation of *MdPG1* by transgenesis in apple reduced pectin depolymerisation and fruit softening (Atkinson et al., 2012). *MdPG1* expression increased rapidly during fruit ripening in the cortex of the short‐shelf‐life Golden Delicious (Li et al., 2010). The expression of *MdPG1* was very weak in cultivars that retain their firmness after long‐term cold storage irrespective of flesh ethylene production (Wakasa et al., 2006). Relatively low MdPG and MdPME activities during early fruit developmental stages were associated with increased cell adhesion and reduced fruit softening rate in the slow‐softening ‘Scifresh’ cultivar (Ng et al., 2013). The expression of an α‐L‐arabinofuranosidase gene (*MdAF3*) was consistently lower in nonmealy genotypes than in those that are mealy (Ng et al., 2013). Moreover, α‐AFase activity was significantly and negatively correlated with fruit firmness but positively correlated with mealiness during a 2‐mo cold storage (Nobile et al., 2011). Another class of cell‐wall modifying proteins, called expansins, has also been associated with fruit softening. Expansins are thought to be involved in the disruption of the noncovalent bonds between the hemicellulose matrix and cellulose microfibrils, thus exposing these polymers to the action of other cell‐wall degrading enzymes (Cosgrove, 1997, 2000). The apple expansin gene *MdExp7* is linked with fruit softening (Costa et al., 2008).

Retention of apple flesh firmness is significantly linked to slower ripening and softening (Marondedze & Thomas, 2012). However, a firmer initial texture does not always correlate with longer shelf life or increased cold storability (Costa et al., 2010). Notably, the mechanisms responsible for fruit firmness or crispness retention are not well understood, both during long‐term cold storage and at room temperature. In this study, bulked segregant analysis via sequencing (BSA‐seq) was employed to identify QTL for apple fruit room‐temperature shelf life. From a major QTL, a pectin actylesterase (*MdPAE10*) was identified and the functional variations were validated.

**Core Ideas**

- Thirteen confident QTL associating apple fruit shelf life were identified.
- A QTL‐derived *MdPAE10* negatively affects fruit shelf life by enhancing pectin solubilization.
- A 379‐bp deletion in the coding sequence of *MdPAE10* disrupts its function.

## 2 MATERIALS AND METHODS

### 2.1 Materials

Bulked segregant analysis via sequencing and QTL identification were performed on 1,273 8‐yr‐old F_1_ individuals between *M. asiatica* Nakai ‘Zisai Pearl’ as the maternal parent and Golden Delicious as the paternal parent. Another segregating population resulting from a cross between Zisai Pearl and Red Fuji containing 1,306 F_1_ individuals, was employed to validate the role of allelic variations in fruit shelf life. Plants were grown at a density of 2.5 by 0.5 m under conventional management and pest control in the Fruit Experimental Station, China Agricultural University (Changping District, Beijing, China).

In 2015–2017, fruit samples were harvested at commercial maturity stage based on fruit characteristics such as starch degradation (selected at 7 on a scale of 1–10) and skin background color (Blanpied & Silsby, 1992). After harvest, apple fruits were stored at room temperature (25–30 °C) with ∼95% relative humidity in polyethylene bags. Flesh firmness and crispness of each F_1_ individuals were measured at 0, 2, 4, and 6 wk after harvest with three biological replicates and three apple fruits per replicate.

To test whether *MdPAE10* expression responded to ethylene signaling, apple fruits (Golden Delicious), prior to commercial ripening (140 d after full bloom), were collected and immediately treated with ethephon or 1‐methylcyclopropene (1‐MCP) as described by Tan, Li, and Wang (2013). The fruit were then stored at room temperature for 20 d. Unripe apple of Golden Delicious were also used for transient transformation experiments.

### 2.2 Methods

#### 2.2.1 Measurement of fruit ethylene production and flesh firmness and crispness

Flesh firmness and crispness were measured using a computer‐controlled texture analyzer TAXT (fruit tester, Stable Micro Systems) as described by Costa et al. (2011, 2012). The diameter of the penetrating probe was 0.2 cm, and the penetration depth was 0.5 cm. The firmness or crispness readings were expressed in N (m kg s^−2^). Three trigonal symmetric punctures were performed on the maximum equator of each apple.

Because of alternate bearing, year‐repeated phenotype data of 403 and 404 F_1_ plants for flesh firmness and flesh crispness, respectively, were obtained for broad‐sense heritability estimation in 2015–2017. The broad‐sense heritability was calculated using the formula, *H* ^2^ = (σ^2^ − σ_e_^2^)/σ^2^, where σ^2^ is the overall phenotype variance, σ_e_^2^ is the environmental variance, that is, the variance among years (Hallauer et al., 1988).

Each fruit was weighed and then enclosed in a gas‐tight container and kept for 2 h at room temperature. One milliliter of gas was sampled from the headspace in the container using a BD syringe (No. 309602, Becton, Dickinson and Company). The ethylene concentration of gas samples was measured with a gas chromatograph HP 5890 series II (Hewlett‐Packard) equipped with a flame ionization detector. Before the gas samples were assayed, the gas chromatograph was calibrated with ethylene gas (NO. 34489, Restek Corporation) at a series of concentrations (0.01, 0.1, 0.5, 1, 5, 10, and 100 mg L^−1^). The fruit ethylene production was calculated with the following formula: *E* = *C* × (*V*_1_ − *V*_2_)/*w*/*T*, where *E* is the fruit ethylene production rate (μl g^−1^ fresh weight h^−1^); *C* is ethylene concentration (mg L^−1^); *V*_1_ indicates the volume of the container (mL); V_2_ is the volume of fruit (mL) equivalent to fresh weight (*w*) in grams, and *T* represents the time (h) kept in the container (Dougherty et al., 2016).

#### 2.2.2 Validation of known *MdACS1* and *MdACO1* markers

The *MdACS1* marker was developed by Sunako et al. (1999). The polymerase chain reaction (PCR) products were *MdACS1‐1/1* (489 bp) and *MdACS1‐1/2* (655 bp). A polymorphism of a 62‐bp insertion–deletion (InDel) in the third intron of *MdACO1* was used to generate the *MdACO1* marker. *MdACO1*‐1/1 and *MdACO1*‐2/2 represented fragment size of 525 and 587 bp, respectively (Costa et al., 2005). Fresh young leaves (100 mg) of the F_1_ individuals or their parental cultivars were used to extract genomic DNA using the modified hexadecyltrimethylammonium bromide (CTAB) protocol described by Li et al. (2013). The PCR reactions were performed and the products were visualized using agarose gel electrophoresis (Jia et al., 2018).

#### 2.2.3 BSA‐seq and candidate gene prediction

Consumers generally accept apple fruit as firm when the flesh firmness >2.2 N; likewise the apple fruits with >0.22 N crispness are referred to as crisp (Costa et al., 2010, 2011; McKay et al., 2011; Nybom et al., 2012). We therefore defined firmness retainability as the maximum number of weeks an apple retains a firmness >2.2 N during room‐temperature storage. By the same way, the number of weeks an apple retains >0.22 N flesh crispness was defined as flesh crispness retainability. The least retainability of flesh firmness or crispness was considered as the room‐temperature shelf life.

To constitute the long shelf life bulk, 30 F_1_ plants from Zisai Pearl × Golden Delicious, which exhibited 6‐wk fruit shelf life, were selected. Conversely, the short shelf life bulk was comprised of 30 F_1_ individuals with 0‐wk shelf life, that is, the flesh firmness was <2.2 N and crispness <0.22 N just after harvest. To reduce the potential contribution of *MdACS1* genotype to the phenotype of fruit shelf life, these 60 F_1_ plants for BSA‐seq analysis were confirmed to have the same *MdACS1‐1/2* genotype because apple cultivars with *MdACS1‐1/2* genotype produced relatively lower levels of ethylene and had longer fruit shelf life than those with *MdACS1‐1/1* genotype. Genomic DNA from each F_1_ individual was extracted from young leaves and pooled to construct segregant bulks. Each bulked DNA sample and DNA of one parent, Zisai Pearl, were sequenced to give 30× genome coverage using a paired‐end 150‐bp read strategy (Illumina X10). The pollen parent, Golden Delicious, has been resequenced previously (Shen et al., 2019).

The resequencing data were processed, and QTL were identified using the BSATOS software package (Shen et al., 2019). Burrows–Wheeler Aligner was used to map clean reads to the apple genome (Li & Durbin, 2009), whereas the GDDH13 genome was used as reference (http://www.rosaceae.org.) (Daccord et al., 2017). Default parameters were used for reads mapping, genotyping, and filtering, and only uniquely mapped reads with a minimum phred‐scaled map quality score of 20 were kept for further analysis. SAMtools was used to identify SNPs and InDels between the aligned sequencing reads and the reference genome. (Li & Durbin, 2009; Li., 2011). To predict the possible function of the variations, genes were annotated. The annotation was performed with ANNOVAR based on the functional domain at the coding sequence (CDS) or the *cis*‐elements at the promoter (Rombauts et al., 1999; Wang & Hakonarson, 2010). A modified *G* value method was used for the statistical analysis of allelic variations between the two bulks (Magwene et al., 2011). By combining the BSA‐seq data and the parental resequencing data, biallele SNPs were categorized into three subsets: *G*‐type, *Z*‐type, and *H*‐type. The *Z*‐type SNPs meant that the SNP genotype of the maternal parent, Zisai Pearl, was heterozygous, while the SNP genotype of the pollen parent, Golden Delicious, was homozygous. Alternatively, *G*‐type SNPs indicated that the SNP genotype was homozygous in Zisai Pearl but was heterozygous in Golden Delicious. The *H*‐type SNPs were those SNP genotypes that were heterozygous in both parents. Quantitative trait loci mapping was run three times with *G*‐type, *Z*‐type, and *H*‐type SNP subsets, and the slide window was set as 1 Mb (Shen et al., 2019). The allele combinations of markers for *G*‐type, *Z*‐type, and *H*‐type QTL were heterozygous in only paternal, maternal, or both parents, respectively. Thus the names of the QTL were initiated with *G*, *Z*, or *H*, respectively.

The genes from QTL interval were downloaded from the apple genome GDDH13 (https://www.rosaceae.org/) (Daccord et al., 2017). By comparing parental resequencing data, genes with identified genetic variations on the parental cultivar on which the QTL was not mapped were removed from the list (Shen et al., 2019). Of these genes, those with SNPs or InDels that did not affect the *cis*‐element on the upstream sequence or the functional domain on the coding region were excluded. For the remaining genes, the UniProt database (http://www.UniProtuniprot.org/) was used to assess the function of the corresponding proteins. Genes that were clearly not related to the target trait were excluded. The pipeline for candidate prediction has been published previously (Liu et al., 2020; Shen et al., 2019).

#### 2.2.4 Relative gene expression assay using quantitative reverse transcription PCR

The complete DNA and complementary DNA (cDNA) sequences of coding region of *MdPAE10* were cloned, Sanger sequenced, and sequence compared between Zisai Pearl and Golden Delicious. The three‐dimensional structure of *MdPAE10* peptide was analyzed by using online tools (https://swissmodel.expasy.org). The primer pairs of *MdPAE10* for quantitative reverse transcription PCR were designed using Primer 5.0 (Premier, Canada). The primer sequences were listed in Supplemental Table S1.

Total RNA was extracted from apple flesh tissue as previously described (Hu et al., 2016). Complementary DNA was synthesized from total RNA using a cDNA Synthesis Kit. Quantitative reverse transcription PCR was carried out as previously reported (Jia et al., 2018). *EF1a* (Loc103447856) was used for internal reference (Jia et al., 2018) (Supplemental Table S1).

#### 2.2.5 KASP assay

To validate the identified variations in *MdPAE10*, 100‐bp upstream and downstream sequences flanking the SNPs were used for the development of Kompetitive allele‐specific PCR (KASP) markers (Chen et al., 2011). The KASP primers are listed in Supplemental Table S1. The KASP assay was performed using the LGC SNPline (LGC Genomics, Laboratory of the Government Chemist) and the data were analyzed using SNPviewer software (LGC).

#### 2.2.6 Vector construction and transient transformation of apple

To determine the impact of InDel2 (a 379‐bp deletion) in the *MdPAE10* CDS on fruit shelf life, the complete *MdPAE10* cDNA sequence containing InDel2 (*MdPAE10‐D*) was amplified from Zisai Pearl. The intact *MdPAE10* CDS without InDel2 (*MdPAE10*) were amplified from Golden Delicious. These segments were cloned into the *Kpn*I and *BamH*I sites, respectively, of the pRI101 vector. The empty pRI101 was used as a control. The pRI101 vector already contains a 35S promoter and a green fluorescent protein reporter gene. The primer pairs used are listed in Supplemental Table S1. The three vectors were transiently transformed into immature Golden Delicious fruit (140 d after full bloom) by using the method described previously (Li et al., 2017). The inoculation sites were recognized by the slightly watery lesions. Flesh texture was measured by penetration at 3–5 mm away from the inoculation sites. The assays were performed with three biological replicates and six apple fruits per replicate per vector.

To test whether the allelic variations in the upstream *MdPAE10* region may affect its promoter activity, the promoter fragments of *MdPAE10.1* (containing InDel1 and SNP1) and *MdPAE10‐D* (containing SNP2) were amplified from genomic DNA of Zisai Pearl. The intact *MdPAE10* promoter segment was amplified from Golden Delicious. All these fragments were sequence compared and digested with *BamH*I/*Nco*I. The fragments were then cloned into the pCAMBIA1391‐GUS (β‐glucuronidase) plant transformation vector as previously reported (Jia et al., 2018). Thus, the three constructs were *pro‐MdPAE10::GUS* (intact); *pro‐MdPAE10.1::GUS* (containing InDel1 and SNP1), and *pro‐MdPAE10‐D::GUS* (containing SNP2). Both *35S::GUS* and the empty pCAMBIA1391‐GUS vector were used as controls. The resulting constructs were transformed into leaf epidermal cells of 4‐wk‐old tobacco (*Nicotiana benthamiana* Domin) plants by vacuum infiltration with *Agrobacterium tumefaciens* strain GV3101.

To test whether variants of the *MdPAE10* promoter respond to ethylene, a 1,000 mg L^−1^ aqueous ethephon solution was sprayed onto the surface of the transformed tobacco leaves 48 h after infiltration. After 2–3 d of transfection, the transgenic leaves were stained using the GUS reporter gene staining kit (Solarbio) in three biological replicates as described by Zhang et al. (2016). Four technical replicates were performed. All primer sequences for vector construction are listed in Supplemental Table S1.

#### 2.2.7 Virus induced gene silencing

A 622‐bp *MdPAE10* CDS fragment was cloned from Golden Delicious into the *Xba*I/*Kpn*I site of the pTRV2 virus vector as previously described (Li et al., 2017). *Agrobacterium tumefaciens* (GV3101) cells harboring the resultant plasmids were suspended in infiltration buffer supplemented with 150 mM acetosyringone. The inoculum preparations were adjusted to OD 600 = 1.0. The mixed vectors and *A. tumefaciens* solution were transformed into Golden Delicious apple fruit by infiltration with a vacuum air pump (Hu et al., 2016; Li et al., 2016). The inoculation sites were recognized by the slightly watery lesions. Flesh texture was measured by penetration at 3–5 mm away from the inoculation sites. The assays were performed with three biological replicates and at least six apple fruits per replicate per vector.

#### 2.2.8 Observation of flesh cellular structure

Flesh tissue was fixed with 10% formalin for 24 h at room temperature and subjected to paraffin embedding. Flesh‐tissue‐embedded paraffin blocks were sectioned (25 μm thick), dewaxed, and the cellular structure was observed using confocal microscopy (Jia et al., 2018).

#### 2.2.9 Pectin extraction and fraction assay

Flesh materials were excised on the lesion from transiently transformed apple cortex tissue (∼10 g). An alcohol‐insoluble cell‐wall preparation was made before the extractions were performed. Then, pectins were extracted sequentially with water, trans‐1,2‐diaminocyclohexane‐*N*,*N*,*N*,*N*‐tetraacetic acid (CDTA), and Na_2_CO_3_ to obtain water‐extractable pectin (WEP), chelator‐extractable pectin (CEP), and Na_2_CO_3_–extractable pectin (NEP) fractions, respectively (Fan et al., 2018). The absorbance was read at 530 nm by a UV–visable spectrophotometer (UV‐1800, SHIMADZU). The pectin content was calculated by a standard curve of D‐(+)‐galacturonic acid.

#### 2.2.10 Statistical analysis

Significant differences were analyzed by pairwise comparisons between corresponding genotypes or treatments using the independent samples Student's *t* test (SPSS Statistics 21; IBM).

## 3 RESULTS

### 3.1 Segregation of flesh firmness and crispness and retainability

**FIGURE 1:**
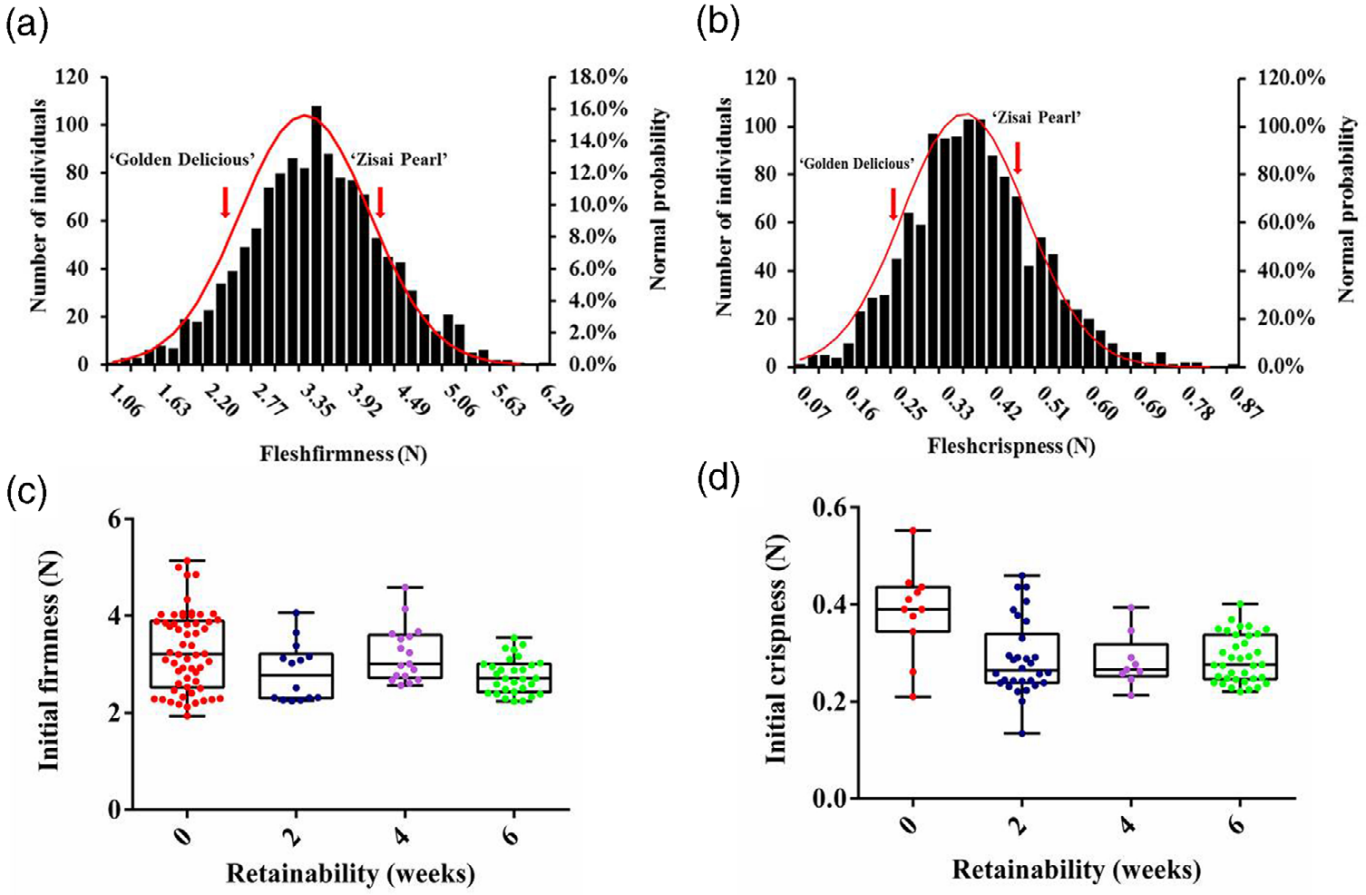
Phenotype segregation for room‐temperature shelf life in an interspecific hybrid population (*Malus asiatica* Nakai ‘Zisai Pearl’ × *M. domestica* Borkh. ‘Golden Delicious’). Frequency distribution of (a) flesh firmness or (b) crispness at harvest. The phenotype values of the parental cultivars were indicated with red arrows. Segregation in (c) flesh firmness retainability or (d) crispness retainability of F_1_ plants with high initial values of flesh firmness and crispness at harvest during room‐temperature storage. One dot in the box plot represents one F_1_ plant

To investigate the segregation of apple flesh firmness or crispness, a collection of 1,273 F_1_ plants derived from the Zisai Pearl × Golden Delicious cultivars were phenotyped. Both initial flesh firmness and crispness immediately after harvest were found to segregate considerably and exhibited Gaussian distribution (*P* < .0001) (Figure 1a, 1b). The broad‐sense heritability of flesh firmness and crispness was 88.68 and 86.12%, respectively. Of the 1,273 F_1_ individuals, the flesh firmness ranged from 1.06 to 6.20 N, while the flesh crispness ranged from 0.07 to 0.87 N. Among them, 1,185 and 1,171 showed high flesh firmness (>2.2 N) and high crispness (>0.22 N) at harvest, respectively, while 1,138 exhibited both high flesh firmness and high crispness. The flesh firmness of Zisai Pearl and Golden Delicious were 4.23 and 2.41 N, respectively. The flesh crispness of the two parental cultivars were 0.47 and 0.25 N, respectively (Figure 1a, 1b).

To measure fruit flesh firmness and crispness retainability, 119 F_1_ plants with both high initial flesh firmness and crispness at harvest were randomly selected for room‐temperature storage. The retainability of flesh firmness and crispness segregated from 2 to 6 wk (Figure 1c, 1d). A significant correlation coefficient (*r* = .2439, *P* < .01) was obtained between flesh firmness and flesh crispness. Finally, 30 and 35 of the 119 F_1_ plants showed high values of flesh firmness (>2.2 N) or flesh crispness (>0.22 N) after 6 wk of room‐temperature storage, respectively (Figure 1c, 1d). The intersection of 30 F_1_ plants with 6 wk of flesh firmness and crispness retainability were referred to as long‐shelf‐life phenotype. Whereas, the F_1_ plants that exhibited both low flesh firmness and low flesh crispness at harvest (0‐wk flesh firmness and crispness retainability) were referred as short‐shelf‐life phenotype. These F_1_ plants were used for subsequent experiments.

### 3.2 Effects of known markers, *MdACS1* and *MdACO1*, on apple fruit shelf life

Many references reported that alleles of *MdACS1*, *MdACS1‐1/1*, *MdACS1‐1/2*, and *MdACS1‐2/2* can result in high, intermediate, and small ethylene production rates in apple flesh (Harada et al., 2000; Oraguzie et al., 2004; Sunako et al., 1999). To determine the effect of *MdACS1* and *MdACO1* genotypes on apple fruit shelf life, the parental cultivars of the hybrid populations were genotyped. The genotype of Zisai Pearl, Red Fuji, and Golden Delicious cultivars was *MdACS1‐1/1:MdACO1‐1/1*, *MdACS1‐2/2:MdACO1‐2/2*, and *MdACS1‐1/2:MdACO1‐1/1*, respectively (Supplemental Figure S1). According to the parental genotypes, *MdACO1* genotype would not segregate in the two F_1_ populations, Zisai Pearl × Red Fuji and Zisai Pearl × Golden Delicious. The *MdACS1* genotype in Zisai Pearl × Golden Delicious population segregated in a 7:9 ratio (*MdACS1‐1/2* : *MdACS1‐1/1*), which is consistent with 1:1 (χ^2^ = 0.25, χ^2^_0.05_ = 7.88) (Supplemental Figure S1; Supplemental Table S2).

**FIGURE 2:**
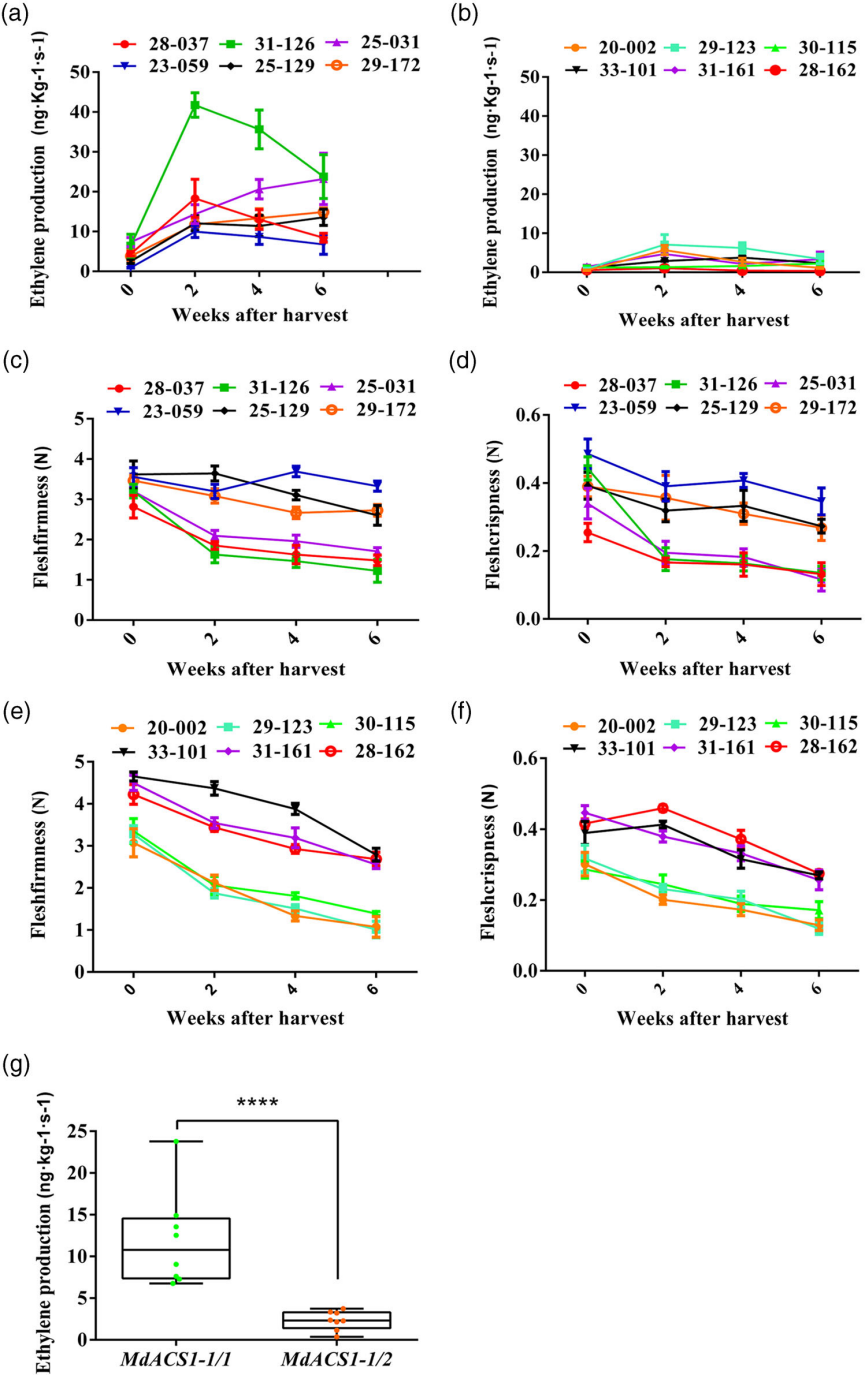
Ethylene production and dynamic changes in flesh firmness or crispness during room‐temperature storage in apple F_1_ plants of *Malus asiatica* Nakai ‘Zisai Pearl’ × *M. domestica* Borkh. ‘Golden Delicious’. Dynamic changes in ethylene production in F_1_ individuals with the (a) *MdACS1‐1/1* and (b) *MdACS1‐2* genotypes during 6 wk of room‐temperature storage. Changes in (c) flesh firmness or (d) flesh crispness in F_1_ individuals with the *MdACS1‐1/1* genotype. Changes in (e) flesh firmness or (f) flesh crispness in hybrids with the *MdACS1‐1/2* genotype. In panels (a) through (f), the error bars represent standard deviation of three biological replicates. (g) Box plot shows the ethylene production rate in individuals with *MdACS1‐1/1* and *MdACS1‐1/2* genotypes. Statistically significant differences were determined by *t* tests (****P < .0001)

The ethylene production rate at harvest, as well as during room‐temperature storage, in fruit with the *MdACS1‐1/2* genotype was significantly lower than that with the *MdACS1‐1/1* genotype (Figure 2a, 2b, 2g). However, the flesh firmness or crispness in the F_1_ plants with the same *MdACS1‐1/1* genotype (28‐037, 25‐031, 31‐126, 23‐059, 25‐129, and 29‐172) segregated during storage as higher in 28‐037, 25‐031, and 31‐126 or as lower in 23‐059, 25‐129, and 29‐172 (Figure 2c, 2d). Completely similar dynamic change in flesh firmness or flesh crispness during storage was observed in F_1_ plants with *MdACS1‐1/2* genotypes (20‐002, 29‐123, 30‐115, 33‐101, 31‐161, and 28‐162) (Figure 2e, 2f). These data indicated that there should be other factors determining fruit shelf life than *MdACS1* genotype.

### 3.3 Identification of QTL for fruit shelf life

**FIGURE 3:**
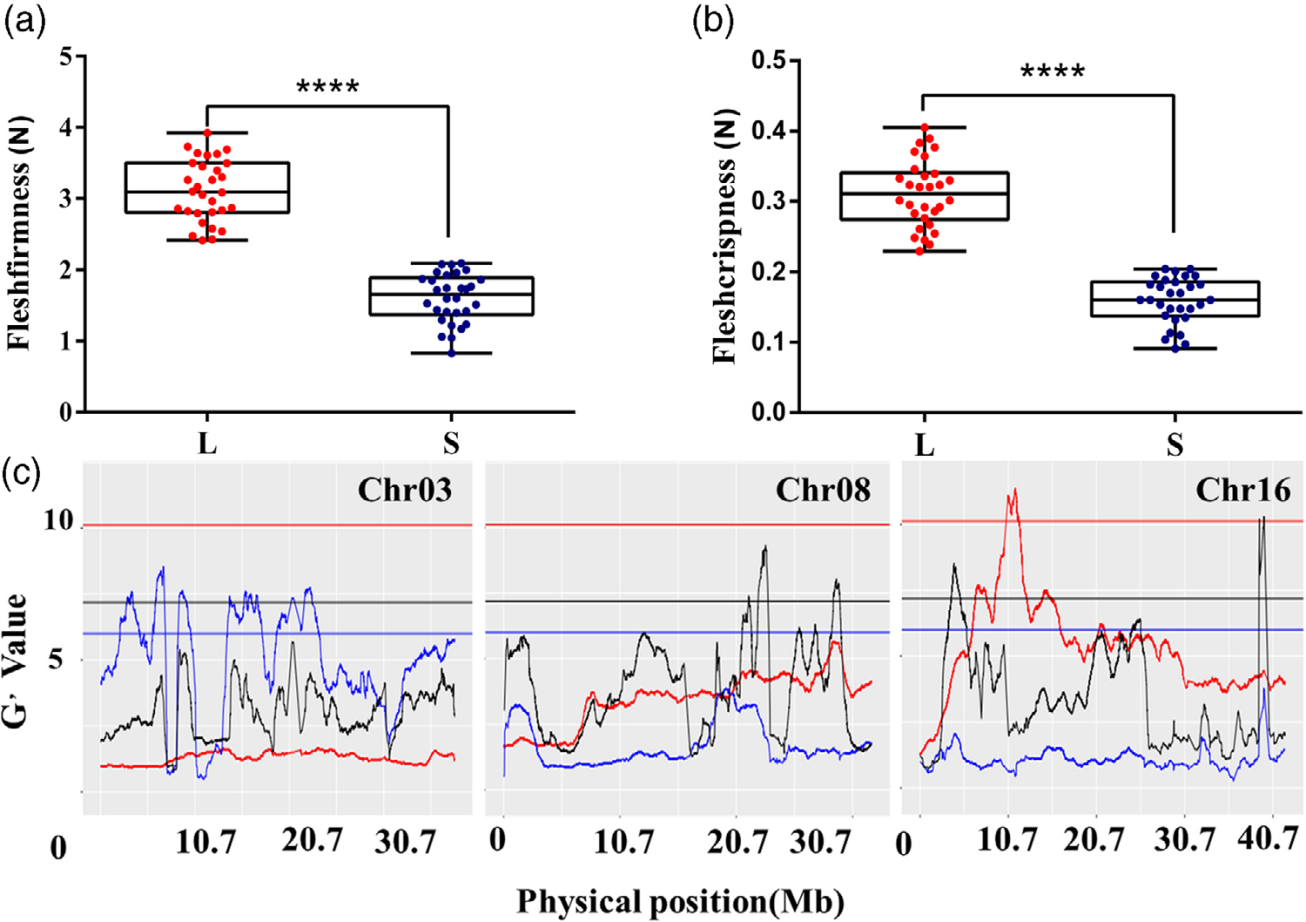
Quantitative trait loci (QTL) associated with apple fruit room‐temperature shelf life identified using a bulked segregant analysis via next‐generation sequencing strategy in an interspecific cross‐population (*Malus asiatica* Nakai ‘Zisai Pearl’ × *M. domestica* Borkh. ‘Golden Delicious’). (a, b) Box plots indicate the composition of the long shelf life (*L*) bulk showing higher flesh firmness and crispness after 6 wk of room‐temperature storage, while the short shelf life (*S*) bulk shows low flesh firmness and crispness at harvest. Statistical significance was determined by the Student's *t* tests between F_1_ individuals with extremely long and short shelf life (*** *P* < .001). (c) Profiles of the significant QTL on chromosome 03 (Chr03), 08 (Chr08), and 16 (Chr16). The solid horizontal lines represent significant thresholds for QTL. The line coloration indicates the parentage of the QTLs (or the thresholds) which were mapped on the maternal (red), paternal (blue) genotypes and both (black) parents

Thirty F_1_ individuals with long shelf life (6 wk) and 30 F_1_ individuals with short shelf life (0 wk) were used to constitute long and short shelf life extremity bulks, respectively (Figure 3a, 3b). The *MdACS1* genotype of all these 60 F_1_ individuals to be used for bulked segregant analysis bulking was *MdACS1‐1*/2. By comparing the resequencing data of Zisai Pearl (unpublished data in our lab) and Golden Delicious (Shen et al., 2019), we identified 10,844,493 SNP markers. These SNPs included 1,823,942 *G*‐type, 7,271,424 *Z*‐type, and 1,749,127 *H*‐type markers between the two parents. Resequencing of the two bulks generated 243,212,629 clean reads (114,767,700 for short shelf life and 128,444,929 for long shelf life). In total, 227,712,155 (93.6%) reads were mapped to the GDDH13 genome. After mapping, genotyping, and filtering, 4,850,112 SNP markers were obtained and the marker densities ranged from 1,324 to 3,661 Mb^−1^. All BSA‐seq raw data have been deposited in the NCBI Sequence Read Archive under the accession number PRJNA546037.

We identified 13 significant QTL for apple fruit shelf life on three chromosomes (Figure 3c; Supplemental Table S3). Seven QTL were mapped to chromosome 03 of Golden Delicious, one QTL was identified on chromosome 16 of Zisai Pearl, and five on chromosome 08 and 16 of both parents (Figure 3c; Supplemental Table S3). Three QTL (H08.2, Z16.1, and H16.2) with *G*´ values >30% of the corresponding thresholds were referred to as major QTL (Supplemental Table S3). The allele effects of nine QTL were positive (δ‐AF > 0) on fruit shelf life, whereas that of four QTL were negative (δ‐AF < 0) (Supplemental Table S4). The contribution of the parents to longer fruit shelf life varied with QTL (Supplemental Table S4).

### 3.4 Candidate gene screening and variation verification

Quantitative trait locus Z16.1 was chosen for candidate gene prediction because Z16.1 exhibited the largest *G*´ value (11.3) (Supplemental Table S3). One hundred thirty‐one genes were annotated in the Z16.1 interval of GDDH13 genome. Of the 131 genes, 17 were removed from the list because the genetic variations occurred in Golden Delicious but not in Zisai Pearl. Of the remaining 114 genes, 82 were selected because the genetic variations of these genes affected the *cis*‐element on the upstream sequence or the functional domain on the CDS (Supplemental Table S5). For the remaining genes, by using the UniProt database, the biological functions of a pectin acetylesterase gene (*MdPAE10*, MD16G1132100) and a pectin lyase‐like superfamily protein gene (*MdPel*, MD16G1140500) are apparently well associated with flesh shelf life (Supplemental TablesS6 and S7). The variations in *MdPAE10*, but not *MdPel*, were confirmed by PCR product Sanger sequencing (Supplemental TablesS6 and S7). *MdPAE10* was used for further variation validation.

**FIGURE 4:**
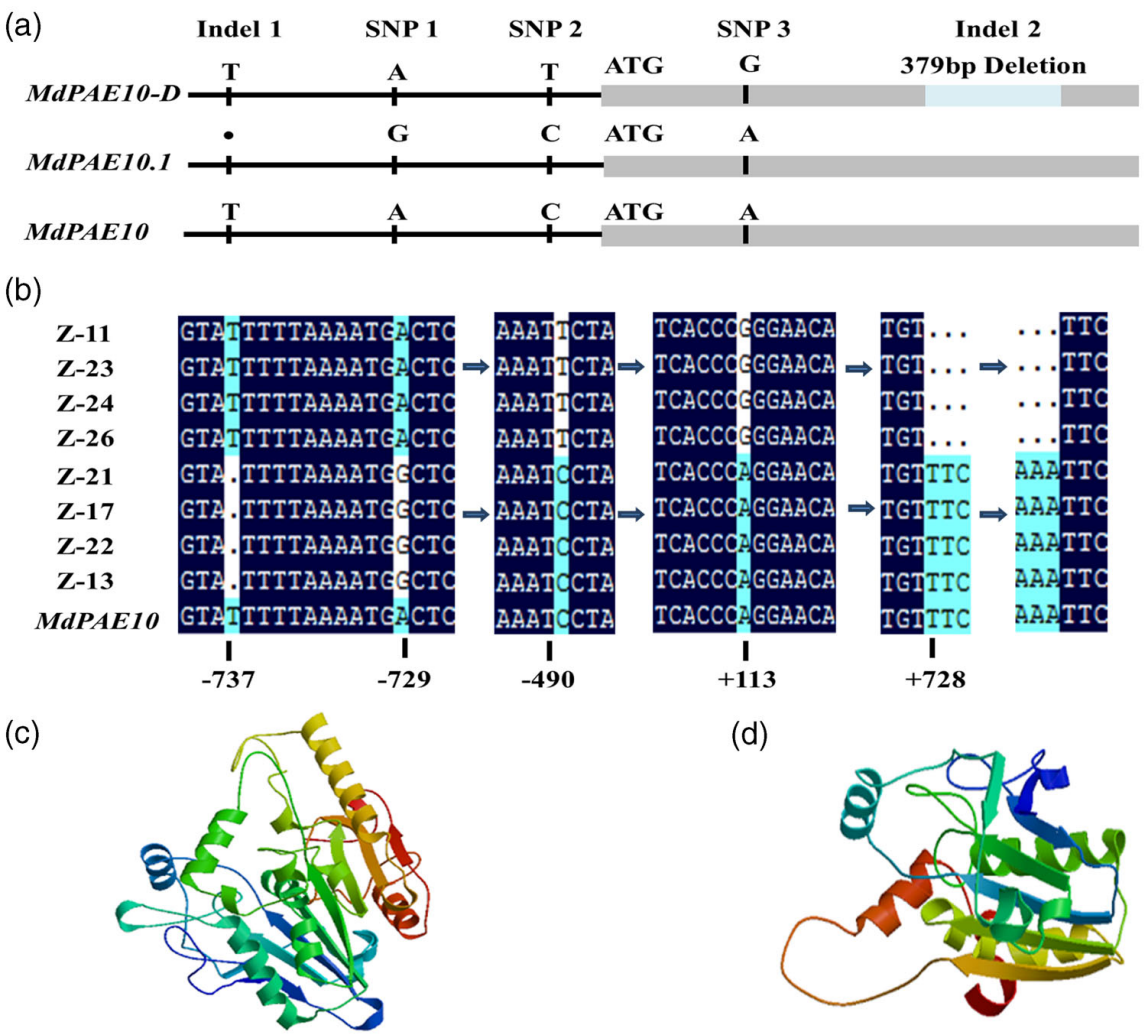
Allelic variations in *MdPAE10* between *Malus asiatica* Nakai ‘Zisai Pearl’ and *M. domestica* Borkh. ‘Golden Delicious’. (a) Diagram showing haplotypes of intact *MdPAE10* in Golden Delicious and *MdPAE10.1* containing SNP1 and InDel1 or *MdPAE10‐D* containing SNP2, SNP3, and InDel2 in Zisai Pearl. The grey rectangle indicates coding sequence and the thick black line shows the upstream region. (b) DNA sequence comparison of the *MdPAE10* coding region and upstream segment of Zisai Pearl (Z) to GDDH13 genome sequence. The variations are located at −737, −729, and −490 bp upstream and +113 and +728 downstream of the ATG codon. (c, d) Bioinformatics predicted three dimensional structures of the intact MdPAE10 protein (c) without the variations and (d) MdPAE10‐D protein containing SNP3 and InDel2

Sanger clone sequencing of *MdPAE10* identified five variations between Golden Delicious and Zisai Pearl (Supplemental Table S8). The variations included three variations at the promoter region, InDel1, T to – at −737 bp; SNP1, A to G at −729 bp; and SNP2, C to T at −490 bp prior to the ATG codon. There were two variations in the CDS. One was SNP3, A to G at +113, which alters a glutamine (Gln) to an arginine (Arg). The other was InDel2, a 379‐bp nucleotide deletion at +728 downstream of the ATG codon (Figure 4a, 4b; Supplemental Table S8). The three‐dimensional structure of the MdPAE10 peptide was predicted to be altered as a consequence of the SNP3 and the InDel2 (Figure 4c, 4d). The SNP2, SNP3, and the InDel2 of *MdPAE10* exhibited complete linkage in the parental cultivars and the F_1_ individuals (Supplemental Table S9).

### 3.5 Validation of the relationship between allelic variation in *MdPAE10* and fruit shelf life

**FIGURE 5:**
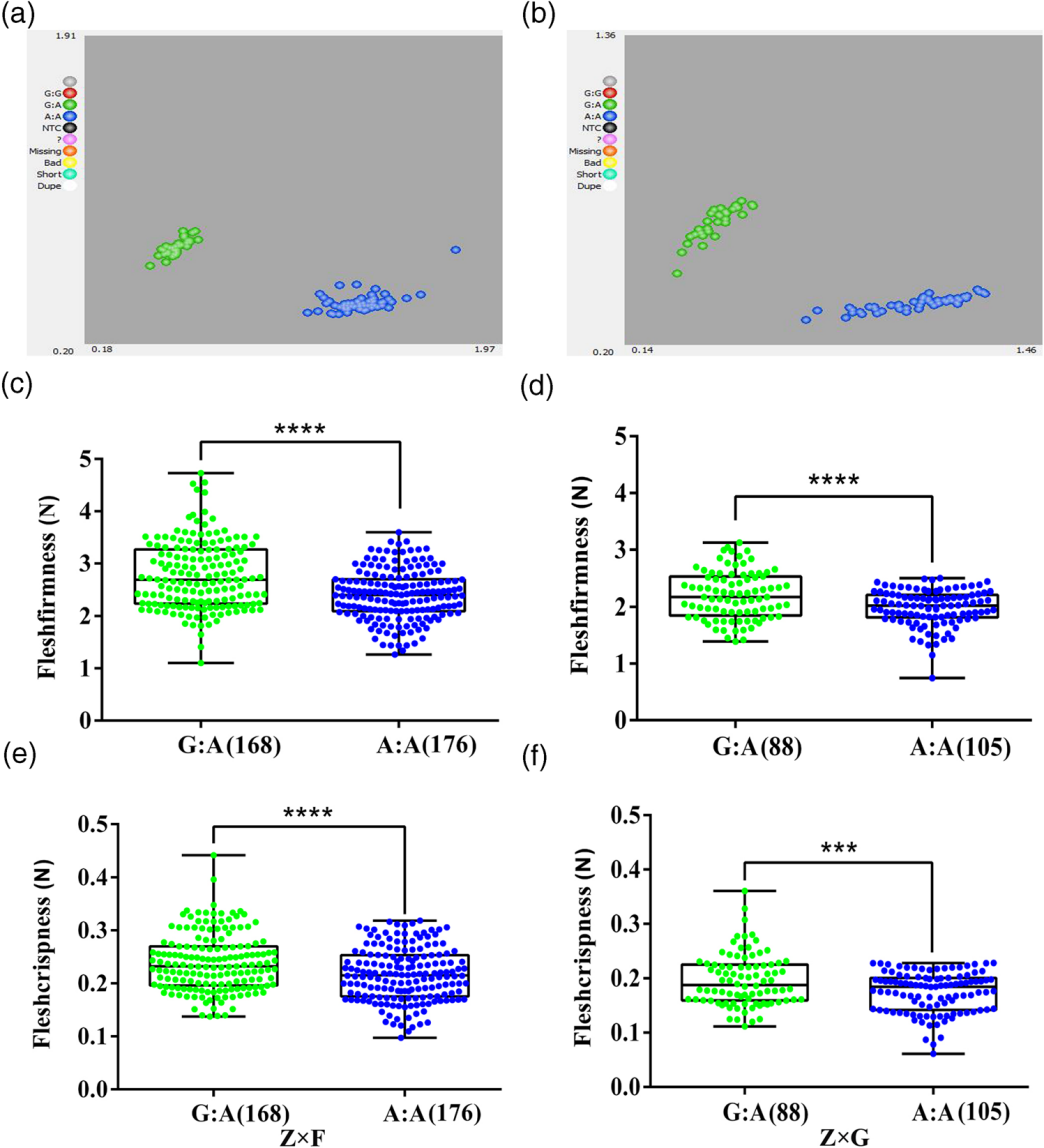
Association between the allelic *MdPAE10* SNP3 (G/A at 113 bp downstream from the ATG codon) genotypes and fruit shelf life in apple cross populations. (a, b) Kompetitive allele‐specific polymerase chain reaction (KASP) genotyping of F_1_ plants of ‘Zisai Pearl’ ×‘Red Fuji’ (left) and Zisai Pearl × ‘Golden Delicious’ (right) showing a 1:1 segregation. (c–f) Comparison of flesh firmness or crispness after 6 wk of room‐temperature storage between genotypes G:A and A:A in F_1_ plants of Zisai Pearl (Z) ×Red Fuji (F) and Zisai Pearl × Golden Delicious (G). Statistical significance was determined by the Student's *t* tests between F_1_ individuals with different genotypes (*P < .05; **P < .01; ***P < .001)

To determine the association of the allelic variations in *MdPAE10* with fruit shelf life, *MdPAE10* SNP3 of 537 (344 from Zisai Pearl × Red Fuji and 193 from Zisai Pearl × Golden Delicious) randomly chosen F_1_ plants were genotyped (Figure 5a, 5b). Chi‐square test revealed a 1:1 segregation between *MdPAE10* SNP3 A:A and G:A genotypes in both of the two F_1_ populations (χ^2^ = 0.19; 0.75, χ^2^_0.05_ = 7.88) (Figure 5c–f). After 6 wk of room‐temperature storage, flesh firmness or crispness of the apple fruits with *MdPAE10* SNP3 A:A genotype were significantly lower than that of the G:A genotype from both populations (Figure 5c–f). These data confirmed the association between allelic variation SNP3 in *MdPAE10* and the phenotype of fruit shelf life. Owing to the tight linkage among SNP2, SNP3, and InDel2, all of the three allelic variations exhibited close association with room‐temperature shelf life (Supplemental Figure S2; Supplemental Table S9).

### 3.6 *MdPAE10* expression pattern and promoter activity

**FIGURE 6:**
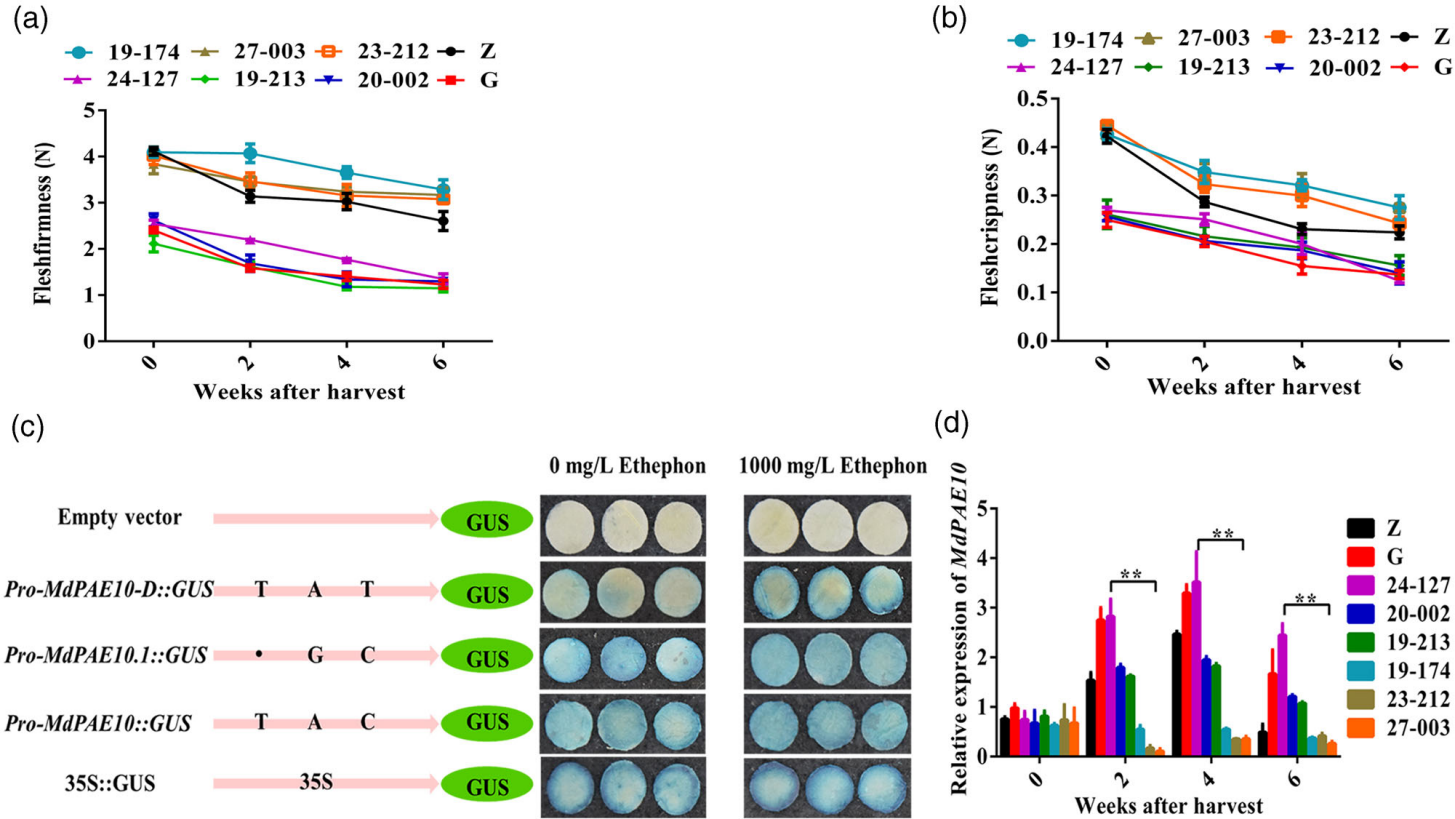
*MdPAE10* expression pattern and promoter activity analysis in F_1_ plants with long (19‐174, 27‐003, and 23‐212) and short shelf life (24‐127, 19‐213, and 20‐002) derived from a cross between *Malus asiatica* Nakai ‘Zisai Pearl’ (Z) × *M. domestica* Borkh. ‘Golden Delicious’ (G). Changes in (a) flesh firmness or (b) crispness of F_1_ plants during room‐temperature storage. (c) Promoter activity assay of *MdPAE10* variants using transiently transformed tobacco leaves and treatment with ethephon before staining. The leaf discs were stained using β‐Glucuronidase (GUS) Reporter Gene Staining Kit. The three horizontally placed leaf discs represent three replicates. (d) *MdPAE10* expression profiles in F_1_ plants with different flesh firmness and crispness retainability and their parents during room‐temperature storage. Error bars indicate standard deviation of three biological replicates, each containing at least three technical replicates. *EF1a* (Loc103447856) was used as the internal reference. Statistical significance was compared between individuals with long and short shelf life by Student's *t* tests (**P < .01)

To investigate whether *MdPAE10* expression differs between the long and short shelf life F_1_ individuals, *MdPAE10* expression were analyzed using three F_1_ plants with long shelf life (19‐174, 27‐003, and 23‐212 with SNP2 C:T/SNP3 A:G/InDel2 Ins:Del genotype) and three F_1_ plants with short shelf life (24‐127, 19‐213, and 20‐002 with SNP2 C:C/SNP3 A:A/InDel2 Ins:Ins genotype) (Supplemental Table S9). The changes in flesh firmness and crispness during room‐temperature storage are shown in Figures 6a and 6b. The *MdPAE10* expression was significantly and consistently lower in the long‐shelf‐life F_1_ individuals and Zisai Pearl than that in the short‐shelf‐life F_1_ individuals and Golden Delicious during 2–6 wk of storage (Figure 6d). The data indicated that the high *MdPAE10* expression was associated with short fruit shelf life, which was possibly attributed to the promotor variations in *MdPAE10*.

Then, to test the effects of variations in *MdPAE10* promoter on the transcription activity, tobacco leaves were transiently transformed. Transformants of *pro‐MdPAE10::GUS* (intact) and *pro‐MdPAE10.1::GUS* (containing InDel1 and SNP1) exhibited the same GUS staining intensity, indicating that InDel1 and SNP1 did not affect promoter activity. The GUS staining intensity was considerably lower in *pro*‐*MdPAE10‐D::GUS* (containing SNP2) transformants than that of *pro‐MdPAE10.1::GUS* (containing InDel1 and SNP1) or *pro‐MdPAE10::GUS* (intact) (Figure 6c). This suggested that the SNP2‐T allele reduced the *MdPAE10‐D* promoter activity (Figure 6c). When the transformants were treated with 1,000 mg L^−1^ ethephon, an ethylene precursor, the GUS staining was more intensive, indicating that the *MdPAE10* promoter from any of the genotypes responded to ethylene (Figure 6c).

### 3.7 Response of *MdPAE10* expression to ethephon or 1‐MCP treatment

**FIGURE 7:**
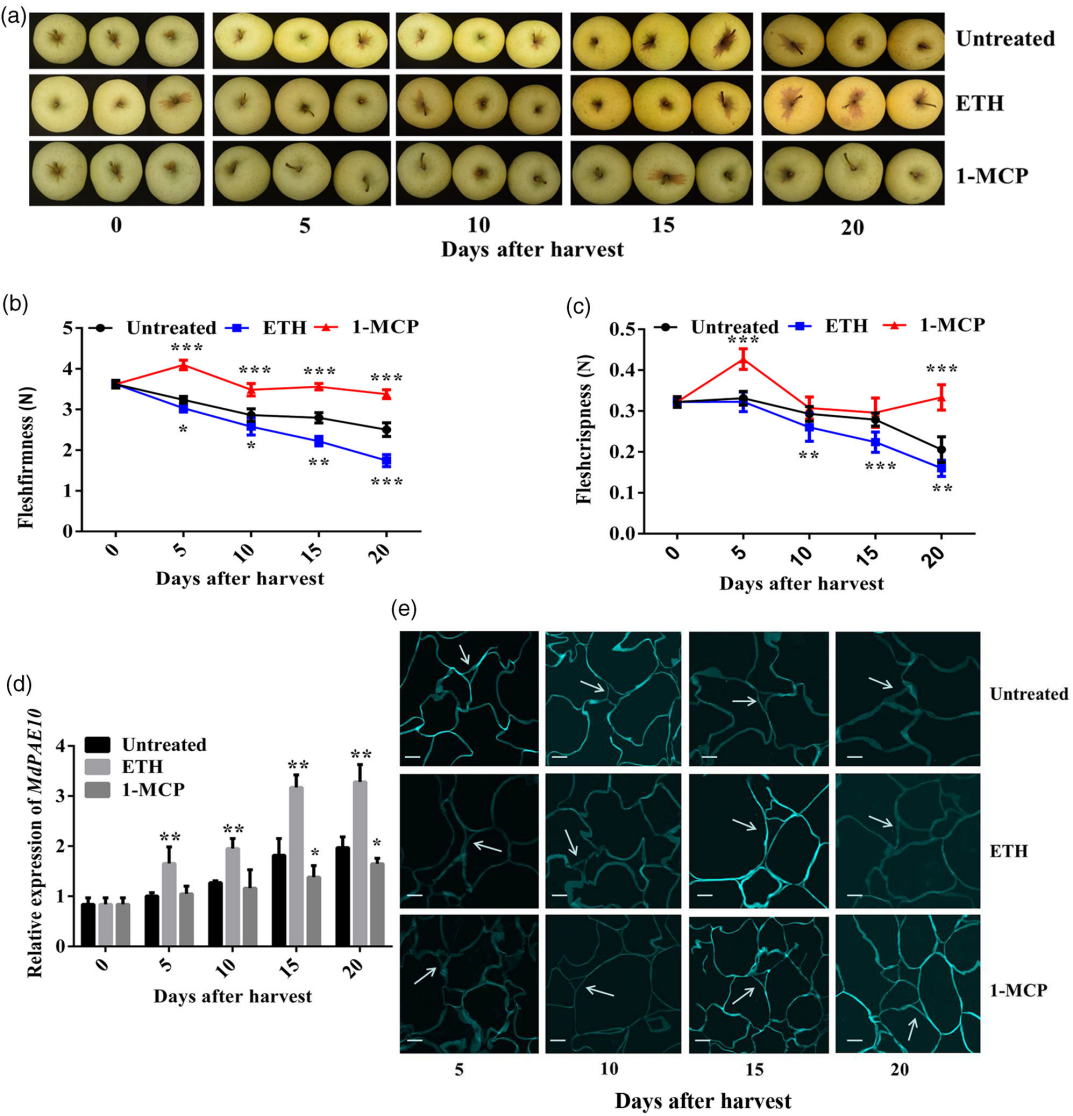
Changes in apple fruit (a) skin color, (b) flesh firmness, (c) crispness, (d) expression of *MdPAE10*, and (e) structure of the parenchyma cells of ‘Golden Delicious’ apple fruits after ethephon or 1‐methylcyclopropene (1‐MCP) treatments. *EF1a* (Loc103447856) was used as the internal reference for (d). Statistical significance in panels (b), (c), and (d) was determined by the Student's *t* tests between treatments at equivalent time points (*P < .05; **P < .01; ***P < .001). Scale bars = 50 μm. The cell wall is pointed out with arrows in (e)

The skin color of Golden Delicious apple fruits typically changes to yellow during ripening and senescence. Here, we observed no obvious changes in fruit skin color in Golden Delicious apple fruits treated with the ethylene inhibitor 1‐MCP during room‐temperature storage. However, the skin color of apple fruits treated with ethephon was more yellow than that of untreated control (Figure 7a). Either flesh firmness or crispness of ethephon treated apple fruits decreased more rapidly during storage than the untreated control, while this decrease was delayed by 1‐MCP treatment (Figure 7b, 7c). *MdPAE10* expression in apple flesh was significantly induced by ethephon treatment at 5–20 d; however, 1‐MCP treatment resulted in significantly lower expression of *MdPAE10* than that in untreated control at 15–20 d (Figure 7d). High fluorescence intensity was observed in apple fruits during 5–10 d storage, demonstrating the tissue structure remained intact (Figure 7e). During 15–20 d of room‐temperature storage in ethephon treatment or nontreated controls, the apple cells became swollen or had irregular morphology (Figure 7e). Also, there was a low fluorescent signal, indicating that pectin fractions in the cell wall were degraded (Figure 7e). In contrast, the cells of 1‐MCP‐treated apple fruits were swollen but were regular in shape and richly fluorescent (Figure 7e). Taken together, both *MdPAE10* expression and flesh firmness or crispness retainability was sensitive to ethylene.

### 3.8 Functional validation of InDel2 in *MdPAE10*

**FIGURE 8:**
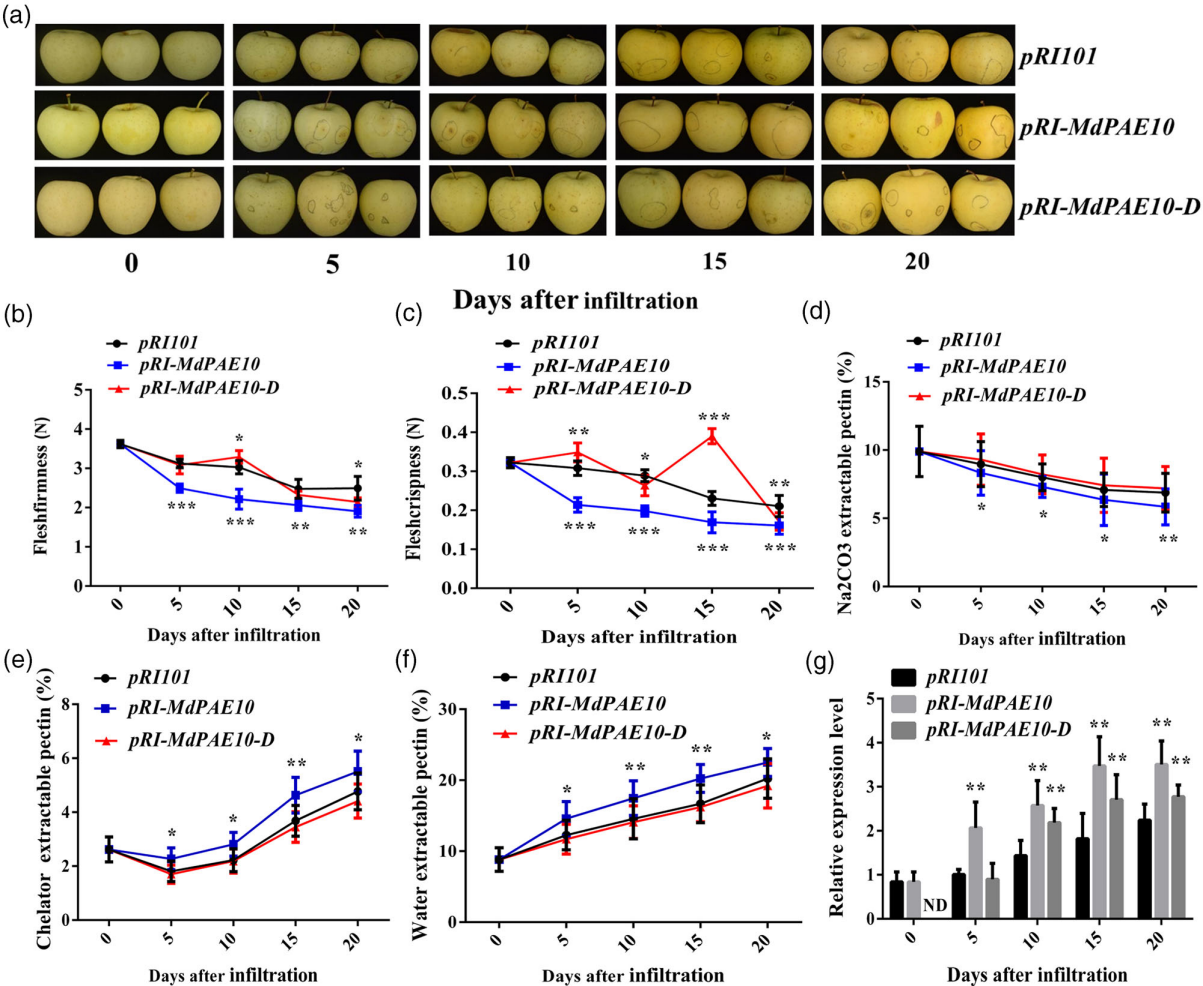
Changes in fruit (a) skin color, (b–f) flesh texture attributes, and (g) *MdPAE10* relative expression in ‘Golden Delicious’ apple fruits transiently overexpression intact *MdPAE10* and *MdPAE10‐D* with InDel2. In panels (b) through (f), all the values were compared with the transformant of pRI empty vector, the statistical significance was marked near the lines. In panel (g), *EF1a* (Loc103447856) was used as the internal reference. Statistical significance was determined by the Student's *t* tests between treatments at equivalent time points (*P < .05; **P < .01; ***P < .001)

The function of InDel2 at *MdPAE10* CDS was validated by using transient transgenesis. A significant increase in *MdPAE10* expression, but no distinct changes in visual fruit skin color, was observed throughout the experiment in apple transiently transformed with *pRI‐MdPAE10‐D* and p*RI‐MdPAE10* than pRI101 empty vector control (Figure 8a, 8g). No robust differences in flesh firmness or flesh crispness were detected between *pRI‐MdPAE10‐D* and empty pRI101‐transformed apple (Figure 8b, 8c). In contrast, both flesh firmness and crispness of *pRI‐MdPAE10*‐transformed apple fruits were significantly lower than that of empty pRI101 control and *pRI*‐*MdPAE10‐D* transformants 5–20 d after transformation (Figure 8b, 8c). Percentage of NEP fraction was relatively lower in *pRI‐MdPAE10*‐transformed apple than that in *pRI‐MdPAE10‐D* and empty pRI101 transformants (Figure 8d). Significantly higher percentages of WEP and CEP fractions were detected in *pRI‐MdPAE10*‐transformed apple than that in empty pRI101 transformants 5–20 d after transformation (Figure 8e, 8c). In *pRI‐MdPAE10*‐*D*‐transformed apple, however, percentage of WEP and CEP was identical with empty pRI101 transformants throughout (Figure 8e, 8f). These data indicated that intact *MdPAE10* functions to accelerate apple flesh softening but that the InDel2 disrupts the function of *MdPAE10*.

**FIGURE 9:**
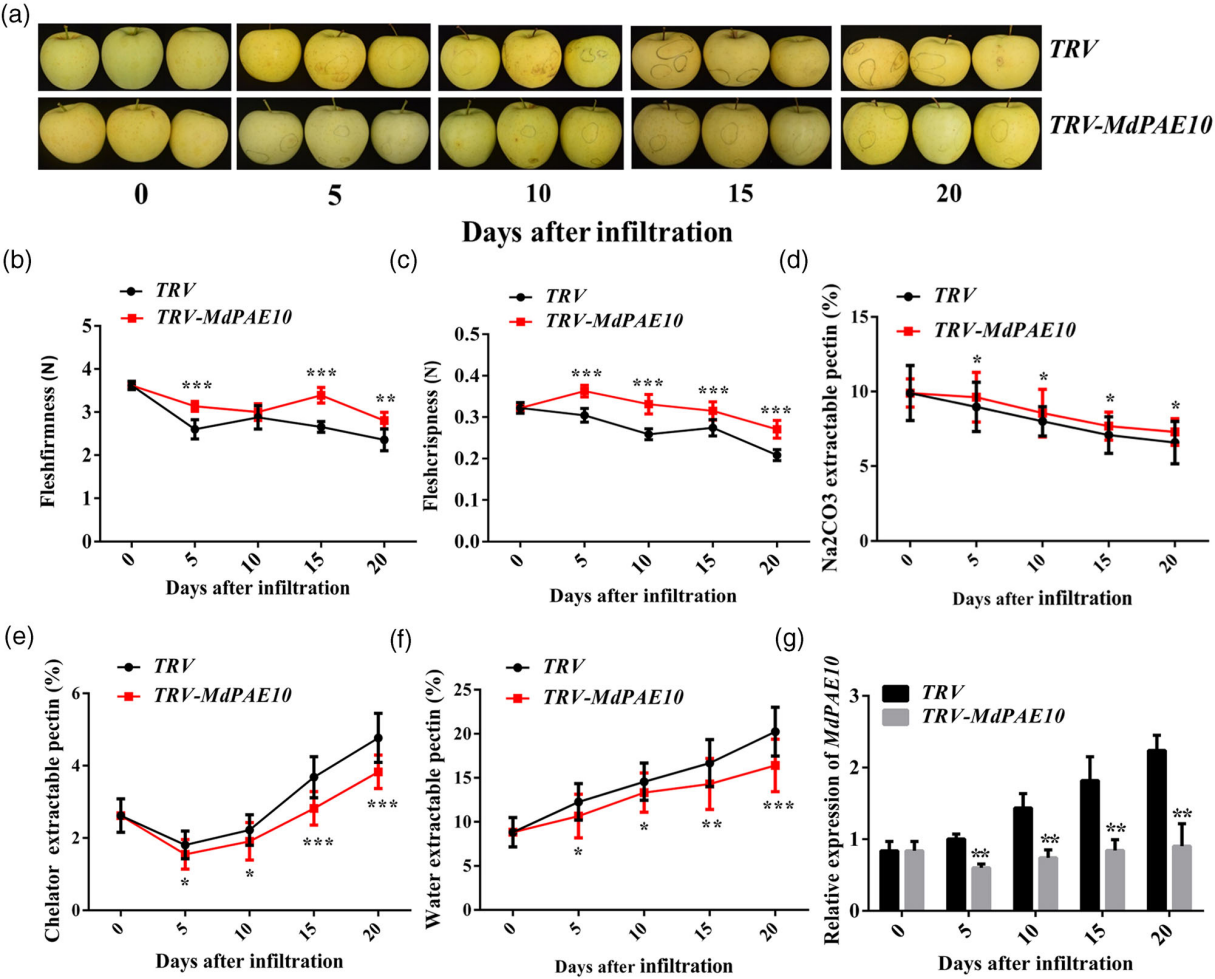
Changes in fruit (a) skin color, (b–f) flesh textural attributes, and (g) *MdPAE10* expression in ‘Golden Delicious’ apple fruits transiently transformed with empty *pTRV* vector or *pTRV‐MdPAE10*. In panels (b) through (f), all the values were compared with the transformant of *TRV* empty vector, the statistical significance was marked near the lines. In panel (g), *EF1a* (Loc103447856) was used as the internal reference. Statistical significance was determined by the Student's *t* tests between treatments at equivalent time points (*P < .05; **P < .01; ***P < .001)

The effect of *MdPAE10* on fruit firmness or crispness retention was further validated by using VIGS. Compared with Golden Delicious apple transiently transformed with an empty *pTRV* vector, apple transformed with *pTRV‐MdPAE10* exhibited slightly less visual yellowing of the skin (Figure 9a). The *MdPAE10* expression was significantly reduced in *pTRV*‐*MdPAE10*‐transformed apple fruits 5–20 d after transformation (Figure 9g). However, significantly higher flesh firmness or crispness was detected in *pTRV*‐*MdPAE10*‐transformed apple 5–20 d after infiltration, except the flesh firmness at day 10 (Figure 9b, 9c). Relatively higher percentage of NEP was observed in *pTRV‐MdPAE10*‐transformed apple than that in the empty TRV control 5–20 d after treatment (Figure 9d). As a consequence, the percentages of WEP and CEP were significantly lower in *pTRV‐MdPAE10* apple than that in empty *TRV*‐transformed control (Figure 9e, 9f). These data further confirmed that *MdPAE10* negatively contribute to flesh firmness or crispness retention by pectin solubilization, hence, silencing *MdPAE10* prolongs apple fruit shelf life.

## 4 DISCUSSION

Room‐temperature shelf life is dominantly determined by the retention of flesh firmness and crispness (Wakasa et al., 2006). The retainability of flesh firmness and crispness is a composite trait dependent on both the initial values of flesh firmness or crispness at harvest and the rate of softening. Most commercial cultivars have high initial flesh firmness and crispness at harvest but the softening rate varied extensively (Nybom et al., 2013). In fact, firmer initial texture does not always correlate with longer shelf life or better firmness or crispness retainability (King et al., 2000, 2001). The larger correlated coefficients between fruit firmness after storage and firmness loss were detected, but that between initial firmness at harvest and firmness loss were often not significant (Chagné et al., 2014). We observed that 93.1 and 91.9% of the F_1_ individuals showed acceptable initial flesh firmness and crispness at harvest, respectively. After 6 wk of room‐temperature storage, however, only 25.2 and 29.3% of the high‐initial‐values F_1_ individuals retained acceptable flesh firmness and crispness. Therefore, on one hand, apple fruit shelf life is correlated with flesh firmness or crispness at harvest, which is consistent with the previous study (Marondedze & Thomas, 2012). On the other hand, low flesh softening rate is more critical for longer shelf life.

Flesh firmness and crispness are important texture attributes for fruit shelf life. From the QTL Z16.1 for fruit shelf life in this study, variations in the candidate gene *MdPAE10* were associated with both flesh firmness and crispness. Another flesh texture attribute, flesh mealiness, was also considered as an index of fruit shelf life. The occurrence of flesh mealiness during storage does not always correspond with the firmness loss or softening (Iwanami, Moriya, Kotoda, Takahashi, & Abe, 2005). A QTL associating apple flesh mealiness has been mapped on chromosome 01 (Kunihisa et al., 2016). However, the interval of the QTL was very near the *MdEXP7* locus, which affects fruit firmness or softening (Costa et al., 2008). We did not measure the degree of flesh mealiness, and no QTL were identified on chromosome 01, which, most likely, is due to the differences in mapping population.

Quantitative trait loci related to fruit shelf life have previously been identified, and chromosome 10 and 15 have been proposed as hot linkage groups (Costa, 2015; Kenis et al., 2008; King et al., 2000; Longhi et al., 2012). In this study, no significant QTL were mapped on chromosome 10 or 15. Instead, we identified 13 confident QTL associating with fruit shelf life on chromosome 03, 08, and 16. Of these QTL, the confident intervals of H16.1 and Z16.1 overlapped with the previously reported QTL at 4.05–4.94 and 9.97–11.27 Mb, respectively (Longhi et al., 2012).

Allelotypes of the functional markers *MdACS1*, *MdACS1‐1/1*, *MdACS1‐1/2*, and *MdACS1‐2/2* have been reported to be associated with high, intermediate, and low ethylene production during fruit ripening (Costa et al., 2005; Harada et al., 2000; Oraguzie et al., 2004; Sunako et al., 1999). Consistently, we observed that fruit of F_1_ plants with *MdACS1‐1/1* genotyepe produced higher levels of ethylene than that with *MdACS1‐1/2* genotype. However, it was not always reliable to predict fruit firmness at harvest or softening rate during cold storage by markers of *MdACS1*, *MdACO1*, *MdEXP7*, or *MdPG1*. These genes accounted for, at maximum, 15% of the observed variation in initial firmness and 18% for softening rate (Longhi et al., 2013; Nybom et al., 2013). For late‐ripening cultivars, *MdACS1‐1/1* genotypes are often firmer at harvest, while the *MdACS1‐2/2* allelotype exhibits a slower rate of flesh softening during cold storage, suggesting that factors other than *MdACS1* contribute substantially to fruit storability (Oraguzie et al., 2007). In this study, the ethylene release rate varied in the *MdACS1‐1/1* and *MdACS1‐1/2* genotypes, while the retainability of flesh firmness or crispness segregated independently of either *MdACS1* genotype or the ethylene emission rate (Figure 2a). By using phenotype‐segregating F_1_ plants with the same *MdACS1‐1/2* genotype, 13 QTL were detected in this study.

A number of cell‐wall metabolism‐related genes, *MdPG1*, *MdPME*, *MdPel*, and Md*Exp7*, promote the degradation of fruit cell walls and therefore accelerate flesh softening (Costa et al., 2008; Longhi et al., 2013; Zhang et al., 2018). We observed that overexpressing intact *MdPAE10*, but not *MdPAE10‐D*, caused a significant decrease in flesh firmness and crispness, whereas VIGS *MdPAE10* led to better flesh firmness and crispness retention. Therefore, *MdPAE10* acts to promote apple flesh softening. The 379‐bp deletion InDel2 disrupts the function of *MdPAE10* and prolongs the fruit shelf life. The less color change in *MdPAE10* VIGS apple was unexpected and the cause was unknown. Besides the InDel2, the two SNPs, SNP2 and SNP3, showed complete genetic linkage with InDel2. The SNP2 in *MdPAE10* promoter leads to a significant reduction in the promoter activity, thus a decrease in *MdPAE10* expression, which also potentially contributes to longer shelf life. The SNP3 at *MdPAE10* CDS altered an amino acid residue, whether or not the SNP3 might contribute to *MdPAE10* function loss is now unclear.

Fruit softening commonly attributes to the loosening of the cell wall, which is related to the degradation of pectin (Bennett & Labavitch, 2008). The NEP is comprised mainly of highly branched rhamnogalacturonan I pectin molecules tightly bound to primary cell wall (Brummell, 2006; Renard, 2005). During the process of cell‐wall loosening, there is generally an increase in soluble pectin and a decrease in insoluble pectin (Song et al., 2016). The action of debranching enzymes, PME, PAE, and PG produces shorter‐chain molecules that are solubilized into CEP and WEP fractions (Gwanpua et al., 2017). The enzyme PAE specifically deacetylates acetylated carbohydrate polymer pectin (Philippe et al., 2017). In this study, transgenesis of *pRI‐MdPAE10* or *pTRV‐MdPAE10* caused only slight changes in the percentage of NEP. However, the percentage of soluble WEP and CEP was increased by *pRI‐MdPAE10* transformation and was significantly decreased by *MdPAE10* VIGS. Such changes of pectin fractions lead to the loss of cohesion of pectin gel matrix, cell‐wall dissolution and cell separation (Xie et al., 2017). As a consequence, the flesh firmness and crispness loss occurred more extensively in *pRI‐MdPAE10*‐transformed apple, but higher flesh firmness and crispness were retained in *pTRV*‐*MdPAE10* transformants. Hence the function of *MdPAE10* on pectin degradation and cell‐wall loosening was further confirmed by these data.

In summary, 13 confident QTL for apple fruit shelf life were identified including three major ones (H08.2, Z16.1, and H16.2). The candidate gene predicted in the QTL Z16.1, *MdPAE10*, was found to be associated with apple fruit shelf life. A 379‐bp deletion in the CDS and a C‐to‐T SNP in the promoter region of *MdPAE10* both perturbed the gene function and prolonged fruit shelf life. These results may contribute to further understanding of apple fruit shelf life, and the functional markers may potentially assist apple breeding.

## AUTHOR CONTRIBUTIONS

BW, CJC, and LL performed the lab experiments. FS analyzed the BSA‐seq data and QTL mapping. XW, WYZ, YD, TWa, ZYH, CX, QZ, YW, and TW contributed to the phenotyping. XZZ, ZHH, and XFX designed and supervised the experiments. BW and XZZ wrote the draft and revised the manuscript.

## CONFLICT OF INTEREST

The authors declare that there are no conflicts of interest.

## Supporting information

Supplemental Figure S1. Genotypes of *MdACS1* (A) and *MdACO1* (B) markers in parental cultivars and the segregation of *MdACS1* marker genotypes in ‘Zisai Pearl’ (Z) × ‘Golden Delicious’ (G) population (C). M, DNA ladder 2000; F, ‘Red Fuji’. 1/1, *MdACS1‐1/1*; 1/2, *MdACS1‐1/2*.

Supplemental Figure S2. Gel image illustrates the segregation in *MdPAE10* InDel2 (379 bp deletion in its coding sequence) genotypes in 12 randomly chosen individuals derived from ‘Zisai Pearl’ (Z)× ‘Golden Delicious’ (G). M, DNA ladder. 1/1 represents *MdPAE10* InDel2 Ins:Ins genotype; 1/2 indicates *MdPAE10* InDel2 Ins:Del genotype.

Supplemental Table S1. The primers used for PCR, qRT‐PCR, and KASP.
Supplemental Table S2. Genotypes of *MdACS1* and *MdACO1* alleles in 64 F1 individuals from *Malus asiatica* Nakai ‘Zisai Pearl’ × *M. domestica* Borkh. ‘Golden Delicious’.
Supplemental Table S3. QTLs identification for apple fruit room‐temperature shelf life by using BSA‐seq strategy in an inter‐specific cross population (*Malus asiatica* Nakai ‘Zisai Pearl’ (Z) × *M. domestica* Borkh. ‘Golden Delicious’ (G)).
Supplemental Table S4. Allele effects of QTLs on fruit shelf life and contribution of the parents.
Supplemental Table S5. Candidate gene prediction in QTL Z16.1.
Supplemental Table S6. Information for candidate genes predicted within the interval of QTL Z16.1 associating apple fruit shelf life.
Supplemental Table S7. Validation of single nucleotide polymorphism variations in the candidate genes via PCR product sequencing.

Supplemental Table S8. Sanger sequencing analysis identified variations in *MdPAE10* promoter between ‘Golden Delicious’ and ‘Zisai Pearl’.

Supplemental Table S9. Complete genetic linkage among genotypes of *MdPAE10* SNP2, SNP3, InDel2, and fruit shelf life phenotype in parental cultivars ‘Zisai Pearl’ and ‘Golden Delicious’, and some of their F1 plants.

## Data Availability

The data that support the findings of this study have been deposited in the NCBI Sequence Read Archive (SRA) under the accession number PRJNA546037.
