# Supplementary material for: Natural variations in a pectin acetylesterase gene, MdPAE10, contribute to prolonged apple fruit shelf life
Source: Plant Genome. 2021 Feb 18;14(1):e20084. doi: 10.1002/tpg2.20084 (PMC12806908; doi:10.1002/tpg2.20084)
Supplement: Supplementary file 1 [file TPG2-14-e20084-s003.docx]

**Supplemental Table S1. The primers used for PCR, qRT-PCR, and KASP.**

| **Name** | **Primer sequences** |  |
| --- | --- | --- |
| MdPAE10-F(qPCR) | CTCAACCCGACTTTGTGCTT | qPCR primers |
| MdPAE10-R(qPCR) | TCTGATACCGGAAACCTTGC |  |
| MdPAE10-D-F(qPCR) | TGTCAAAGGGAATGCGTTAT |  |
| MdPAE10-D-R(qPCR) | AAAGCAAGAAACAATCCACC |  |
| MdPAE10-F | CCATTTGTATGCGCAATGAGATTTC | Primers for CDS |
| MdPAE10-R | TCACTTAAACACCAGATTATGGCAGGT |  |
| PRI-MdPAE10-F | GGGGTACCCCATTTGTATGCGCAATGAGATTTC | Primers for OE |
| PRI-MdPAE10-R | CGGGATCCTCACTTAAACACCAGATTATGGCAGGT |  |
| TRV2-MdPAE10-F | GCTCTAGAGATGTCAAAGGGAATGCGTTATGCT | Primers for VIGS |
| TRV2-MdPAE10-R | GGGGTACCTCCGACTGATTTTGCTATCCCCTTA |  |
| MdPAE10-AlleleX | TGTATAATGCAGGTCTTGTTCCT | Primers for KASP |
| MdPAE10-AlleleY | TGTATAATGCAGGTCTTGTTCCC |  |
| MdPAE10-F(marker) | TCTCATCCTCGCGTTTCTTTCT | Primers for markers |
| MdPAE10-R(marker) | CCGACTGATTTTGCTATCCCCT |  |
| MdPAE10-F(marker promoter) | CAAAGCCGGAGAACCTCAGT |  |
| MdPAE10-R(marker promoter) | GAAGAAAGGAAGGGGGAGGC |  |
| MdPL-F(marker promoter) | GCAGTTGGTGGACTGCAATG |  |
| MdPL-R(marker promoter) | ACGATATTCACCTGGTCGCT |  |
| MdEXPA1-F(marker) | GGCGGTTTGCTAGCACATTT |  |
| MdEXPA1-R(marker) | GGGAGAGGAAATGCGAGGAG |  |
| MdPAE10-F(promoter) | ACAAAATGGGGTGGCTGG | Primers for promoters |
| MdPAE10-R(promoter) | AAGGAAGGGGGAGGCAGAG |  |
| MdPAE10-F(BamH1) | CGGGATCCACAAAATGGGGTGGCTGG | Primers for GUS |
| MdPAE10-R(Nco1) | CATGCCATGGAAGGAAGGGGGAGGCAGAG |  |

**Supplemental Table S2. Genotypes of *MdACS1* and *MdACO1* alleles in 64 F1 individuals from *Malus asiatica* Nakai ‘Zisai Pearl’ × *M. domestica* Borkh. ‘Golden Delicious’.**

| **Number** | ***Md-ACS*1 genotype** | ***Md-ACO*1 genotype** |
| --- | --- | --- |
| 21-031 | 1/1 | 2/2 |
| 22-036 | 1/1 | 2/2 |
| 22-120 | 1/1 | 2/2 |
| 22-161 | 1/1 | 2/2 |
| 23-007 | 1/1 | 2/2 |
| 23-030 | 1/1 | 2/2 |
| 23-059 | 1/1 | 2/2 |
| 23-066 | 1/1 | 2/2 |
| 23-070 | 1/1 | 2/2 |
| 23-154 | 1/1 | 2/2 |
| 24-106 | 1/1 | 2/2 |
| 24-198 | 1/1 | 2/2 |
| 24-201 | 1/1 | 2/2 |
| 24-212 | 1/1 | 2/2 |
| 25-031 | 1/1 | 2/2 |
| 25-129 | 1/1 | 2/2 |
| 26-052 | 1/1 | 2/2 |
| 26-053 | 1/1 | 2/2 |
| 26-209 | 1/1 | 2/2 |
| 27-101 | 1/1 | 2/2 |
| 28-037 | 1/1 | 2/2 |
| 28-057 | 1/1 | 2/2 |
| 28-097 | 1/1 | 2/2 |
| 28-107 | 1/1 | 2/2 |
| 31-126 | 1/1 | 2/2 |
| 31-209 | 1/1 | 2/2 |
| 29-172 | 1/1 | 2/2 |
| 30-004 | 1/1 | 2/2 |
| 30-052 | 1/1 | 2/2 |
| 31-007 | 1/1 | 2/2 |
| 31-126 | 1/1 | 2/2 |
| 32-063 | 1/1 | 2/2 |
| 32-104 | 1/1 | 2/2 |
| 32-108 | 1/1 | 2/2 |
| 32-171 | 1/1 | 2/2 |
| 32-179 | 1/1 | 2/2 |
| 32-179 | 1/1 | 2/2 |

| **Number** | ***Md-ACS*1genotype** | ***Md-ACO*1 genotype** |
| --- | --- | --- |
| 20-002 | 1/2 | 2/2 |
| 21-096 | 1/2 | 2/2 |
| 23-070 | 1/2 | 2/2 |
| 24-005 | 1/2 | 2/2 |
| 25-047 | 1/2 | 2/2 |
| 25-175 | 1/2 | 2/2 |
| 26-010 | 1/2 | 2/2 |
| 26-084 | 1/2 | 2/2 |
| 27-003 | 1/2 | 2/2 |
| 28-016 | 1/2 | 2/2 |
| 28-162 | 1/2 | 2/2 |
| 28-188 | 1/2 | 2/2 |
| 29-039 | 1/2 | 2/2 |
| 29-039 | 1/2 | 2/2 |
| 29-068 | 1/2 | 2/2 |
| 29-123 | 1/2 | 2/2 |
| 29-186 | 1/2 | 2/2 |
| 29-186 | 1/2 | 2/2 |
| 30-031 | 1/2 | 2/2 |
| 30-053 | 1/2 | 2/2 |
| 30-115 | 1/2 | 2/2 |
| 30-145 | 1/2 | 2/2 |
| 31-151 | 1/2 | 2/2 |
| 31-161 | 1/2 | 2/2 |
| 32-113 | 1/2 | 2/2 |
| 32-113 | 1/2 | 2/2 |
| 33-034 | 1/2 | 2/2 |
| 33-101 | 1/2 | 2/2 |

Note: * ACS1（1：489bp/2:655bp）; ACO1（1：525 bp/2：587 bp）.

**Supplemental Table S3. QTLs identification for apple fruit room-temperature shelf life by using BSA-seq strategy in an inter-specific cross population (*Malus asiatica* Nakai ‘Zisai Pearl’ (Z) × *M. domestica* Borkh. ‘Golden Delicious’ (G)).**

| **QTLs*** | **parent** | | | **G^，^Value** | **Peak loacation (Mb)** | **Intervals (Mb)** |
| --- | --- | --- | --- | --- | --- | --- |
| Z16.1 | | Z | | 11.3 | 10.8 | 9.9-11.2 |
| H16.1 | | | Z&G | 7.2 | 4.5 | 4.0-5.0 |
| H16.2 | | | Z&G | 10.3 | 38.9 | 38.4-39.4 |
| G03.1 | | | G | 7.7 | 22.2 | 2.05-2.27 |
| G03.2 | | | G | 6.1 | 4.1 | 3.6-4.6 |
| G03.3 | | | G | 6.3 | 5.5 | 5.0-6.0 |
| G03.4 | | | G | 8.5 | 6.6 | 5.6-7.1 |
| G03.5 | | | G | 7.6 | 8.6 | 8.1-9.5 |
| G03.6 | | | G | 7.6 | 15.3 | 13.4-17.0 |
| G03.7 | | | G | 7.7 | 22.2 | 18.6-23.1 |
| H08.1 | | | Z&G | 7.4 | 21.0 | 20.5-21.5 |
| H08.2 | | | Z&G | 9.3 | 22.5 | 21.8-23.0 |
| H08.3 | | | Z&G | 8.0 | 28.5 | 28.0-29.0 |

Note: * QTLs names are formatted as ‘parentage + chromosome number + serial number’, for examples, G03.3 means the third QTL mapped on chromosome 03 of ‘Golden Delicious’; Z16.1 means the only one QTL identified on chromosome 16 of ‘Zisai Pearl’; while H08.2 represents the second QTL on chromosome 08 of both parents.

**Supplemental Table S4. Allele effects of QTLs on fruit shelf life and contribution of the parents.**

| **QTL** | **Peak position** | **Variation** | **AF-L** | **AF-S** | **δ-AF** | **Genotype (Z)** | **Genotype (G)** | **Parent contribution (Z)** | **Parent contribution (G)** |
| --- | --- | --- | --- | --- | --- | --- | --- | --- | --- |
| Z16.1 | 10,805,133 | C/A | 0.42 | 0.06 | 0.36 | C:A | C:C | Positive | Negative |
| H16.1 | 4,540,924 | C/T | 0.19 | 0.67 | -0.48 | C:T | C:T | Negative | Negative |
| H16.2 | 38,985,473 | C/T | 0.24 | 0.37 | -0.13 | C:T | C:T | Negative | Negative |
| G03.1 | 22,205,258 | C/A | 0.38 | 0.06 | 0.32 | C:C | C:A | Negative | Positive |
| G03.2 | 4,168,844 | T/G | 0.26 | 0.05 | 0.21 | T:T | T:G | Negative | Positive |
| G03.3 | 5,565,352 | G/A | 0.48 | 0.16 | 0.32 | G:G | G:A | Negative | Positive |
| G03.4 | 6,665,517 | C/T | 0.27 | 0.16 | 0.11 | C:C | C:T | Negative | Positive |
| G03.5 | 8,694,543 | G/A | 0.54 | 0.05 | 0.49 | G:G | G:A | Negative | Positive |
| G03.6 | 15,338,493 | C/T | 0.39 | 0.07 | 0.32 | C:C | C:T | Negative | Positive |
| G03.7 | 22,205,258 | C/A | 0.38 | 0.06 | 0.32 | C:C | C:A | Negative | Positive |
| H08.1 | 21,060,394 | T/G | 0.47 | 0.70 | -0.23 | T:G | T:G | Negative | Negative |
| H08.2 | 22,571,166 | G/A | 0.39 | 0.16 | 0.23 | G:A | G:A | Positive | Positive |
| H08.3 | 28,557,233 | A/C | 0.33 | 0.79 | -0.46 | A:C | A:C | Negative | Negative |

Note: Z represents ‘Zisai Pearl’; G, represents ‘Golden Delicious’ . AF-L and AF-S indicate allele frequency in long and short shelf life bulks, respectively. δ-AF means the deviation of allele frequency between long and short shelf life bulks.

| **Supplemental Table S5. Candidate gene prediction in QTL Z16.1.** | |
| --- | --- |
| **Gene name** | **gene annotion** |
| MD16G1130500 | Plant protein of unknown function (DUF247) |
| MD16G1130600 | Protein of unknown function (DUF1624) |
| MD16G1130700 | ASH1-related protein 2 |
| MD16G1130800 | eukaryotic release factor 1-3 |
| MD16G1130900 | U2 small nuclear ribonucleoprotein A |
| MD16G1131100 | Protein of unknown function (DUF1350) |
| MD16G1131200 | Exostosin family protein |
| MD16G1131300 | Other Eukaryotes -339 (source: NCBI BLink) |
| MD16G1131600 | DEA(D/H)-box RNA helicase family protein |
| MD16G1131800 | Galactose oxidase/kelch repeat superfamily protein |
| MD16G1131900 | Alkaline-phosphatase-like family protein |
| MD16G1132000 | nitrate transporter 1:2 |
| MD16G1132100 | Pectinacetylesterase family protein |
| MD16G1132300 | neurofilament protein-related |
| MD16G1132500 | indole-3-acetic acid inducible 31 |
| MD16G1132700 | TRAF-like family protein;function |
| MD16G1133100 | Other Eukaryotes -0 (source: NCBI BLink) |
| MD16G1133300 | Other Eukaryotes -2996 (source: NCBI BLink) |
| MD16G1133600 | Thioesterase superfamily protein |
| MD16G1133900 | Saposin-like aspartyl protease family protein |
| MD16G1134700 | ATP binding cassette subfamily B4 |
| MD16G1135100 | P-glycoprotein 11 |
| MD16G1135200 | 5'-nucleotidase |
| MD16G1135300 | pigment defective 320 |
| MD16G1135500 | Pseudouridine synthase family protein |
| MD16G1135600 | MuDR family transposase;function |
| MD16G1136100 | CBL-interacting protein kinase 1 |
| MD16G1136200 | ribosomal protein L3 plastid;function |
| MD16G1136400 | Plant invertase/pectin methylesterase inhibitor superfamily |
| MD16G1136500 | Tetratricopeptide repeat (TPR)-like superfamily protein |
| MD16G1136600 | hAT dimerisation domain-containing protein |
| MD16G1136700 | Fcf2 pre-rRNA processing protein |
| MD16G1136800 | pyruvate dehydrogenase kinase |
| MD16G1137000 | novel plant snare 13 |
| MD16G1137200 | Protein kinase superfamily protein |
| MD16G1137300 | Protein kinase superfamily protein |
| MD16G1137600 | squamosa promoter-binding protein-like 12 |
| MD16G1137700 | squamosa promoter binding protein-like 1 |
| MD16G1137800 | Tetratricopeptide repeat (TPR)-like superfamily protein |
| MD16G1137900 | pleiotropic drug resistance 9 |
| MD16G1138000 | pleiotropic drug resistance 9 |
| MD16G1138100 | Amino acid dehydrogenase family protein |
| MD16G1138300 | S-adenosylmethionine synthetase 1 |
| MD16G1138500 | TRAF-like family protein |
| MD16G1138700 | TRAF-like family protein |
| MD16G1138800 | TRAF-like family protein |
| MD16G1139000 | signal responsive 1 |
| MD16G1139200 | Calmodulin-binding transcription activator protein |
| MD16G1139300 | phragmoplast orienting kinesin 1 |
| MD16G1139400 | Other Eukaryotes -0 (source: NCBI BLink) |
| MD16G1139600 | 1-cysteine peroxiredoxin 1 |
| MD16G1140200 | S-adenosyl-L-methionine-dependent methyltransferases superfamily protein |
| MD16G1140300 | HCO3 -transporter family |
| MD16G1140400 | Other Eukaryotes -8 (source: NCBI BLink) |
| MD16G1140500 | Pectin lyase-like superfamily protein |
| MD16G1140800 | Integrase-type DNA-binding superfamily protein |
| MD16G1141000 | Other Eukaryotes -3 (source: NCBI BLink) |
| MD16G1141100 | Phosphoglycerate mutase family protein |
| MD16G1141200 | glycyl-tRNA synthetase / glycine--tRNA ligase |
| MD16G1141400 | Sec14p-like phosphatidylinositol transfer family protein |
| MD16G1141600 | Exostosin family protein |
| MD16G1141700 | Aldolase-type TIM barrel family protein |
| MD16G1141800 | FKBP-like peptidyl-prolyl cis-trans isomerase family protein |
| MD16G1141900 | CYTOCHROME P450 51G1 |
| MD16G1142000 | Other Eukaryotes -3 (source: NCBI BLink) |
| MD16G1142100 | Major facilitator superfamily protein |
| MD16G1142200 | RING/U-box superfamily protein |
| MD16G1142300 | transferring glycosyl groups |
| MD16G1142800 | Auxin-responsive GH3 family protein |
| MD16G1143000 | ribosomal protein S27 |
| MD16G1143100 | nudix hydrolase homolog 13 |
| MD16G1143400 | Other Eukaryotes -0 (source: NCBI BLink) |
| MD16G1143900 | high affinity K+ transporter 5 |
| MD16G1144400 | 7 sterol isomerase |
| MD16G1144500 | Calcium-dependent lipid-binding (CaLB domain) family protein |
| MD16G1144600 | Other Eukaryotes -339 (source: NCBI BLink) |
| MD16G1144700 | minichromosome maintenance (MCM2/3/5) family protein |
| MD16G1144800 | calnexin 1 |
| MD16G1144900 | Other Eukaryotes -20 (source: NCBI BLink) |
| MD16G1145000 | SNARE associated Golgi protein family |
| MD16G1145100 | respiratory burst oxidase homologue D |
| MD16G1145200 | xyloglucan endotransglucosylase/hydrolase 5 |

**Supplemental Table S6. Information for candidate genes predicted within the interval of QTL Z16.1 associating apple fruit shelf life.**

| **Chromosome** | **Position** | **Reference** | **Alter** | **Region** | **Gene** | **G** | **Z** | **QTL** |
| --- | --- | --- | --- | --- | --- | --- | --- | --- |
| Chr16 | 10069850 | T | - | upstream | MD16G1132100 | TT | T/. | Z16.1 |
| Chr16 | 10069857 | A | G | upstream | MD16G1132100 | AA | AG | Z16.1 |
| Chr16 | 10070096 | C | T | upstream | MD16G1132100 | CC | CT | Z16.1 |
| Chr16 | 10072940 | G | T | exonic | MD16G1132100 | GG | GT | Z16.1 |
| Chr16 | 10073634 | A | G | exonic | MD16G1132100 | AA | AG | Z16.1 |
| Chr16 | 10073639 | C | A | exonic | MD16G1132100 | CC | CA | Z16.1 |
| Chr16 | 10073641 | A | C | exonic | MD16G1132100 | AA | AC | Z16.1 |
| Chr16 | 10073647 | C | G | exonic | MD16G1132100 | CC | CG | Z16.1 |
| Chr16 | 10073649 | A | C | exonic | MD16G1132100 | AA | AC | Z16.1 |
| Chr16 | 10073650 | A | G | exonic | MD16G1132100 | AA | AG | Z16.1 |
| Chr16 | 10801190 | G | C | exonic | MD16G1140500 | GG | GC | Z16.1 |
| Chr16 | 10801988 | G | C | exonic | MD16G1140500 | GG | GC | Z16.1 |
| Chr16 | 10802548 | G | T | exonic | MD16G1140500 | GG | GT | Z16.1 |
| Chr16 | 10802550 | T | C | exonic | MD16G1140500 | TT | TC | Z16.1 |
| Chr16 | 10806127 | T | C | upstream | MD16G1140500 | TT | TC | Z16.1 |
| Chr16 | 10806134 | G | T | upstream | MD16G1140500 | GG | GT | Z16.1 |

Note: G represents ‘Golden Delicious’, Z represents‘Zisai Pearl’.

**Supplemental Table S7. Validation of single nucleotide polymorphism variations in the cadidate genes via PCR product sequencing.**

| **Gene ID** | | **1** | **2** | **3** |
| --- | --- | --- | --- | --- |
| MD16G1132100 |  | |  |  |
|  | **4** | | **5** | **6** |
|  |  | |  |  |

| **Gene ID** | **1** | **2** |
| --- | --- | --- |
| MD16G1140500 |  |  |
